# A scalable UWB-to-reconfigurable MIMO filtenna with single-varactor tuning and enhanced isolation for adaptive 5G and cognitive radio systems

**DOI:** 10.1038/s41598-026-36882-8

**Published:** 2026-02-13

**Authors:** Hager S. Fouda, Amal S. Hamoud, Mahmoud A. Attia

**Affiliations:** 1 Electronics and Electrical Communications Engineering Department, Tanta University, Tanta, Egypt; 2 Electronics and Electrical Communications Engineering Department, Faculty of Engineering, Horus University-Egypt, New Damietta, Egypt

**Keywords:** Cognitive radio (CR), Fifth generation (5G), Filtenna, Multiple-input-multiple-output (MIMO), Ultra-wideband (UWB), Engineering, Physics

## Abstract

This work presents a complete development framework that begins with a new fork-shaped ultra wideband (UWB) antenna. The antenna is designed, optimized, fabricated, and experimentally validated. The prototype achieves a very wide bandwidth extending from 2.4 to 8 GHz, with stable radiation behavior and high efficiency. Building on this design, a 4 × 4 UWB MIMO array is developed. The four elements are arranged orthogonally to enhance isolation and provide strong pattern diversity. Next, the UWB antenna is transformed into a frequency-reconfigurable filtering antenna (filtenna). A single varactor diode is embedded in a modified radiator to enable continuous tuning from 2.45 to 3.48 GHz. A stepped ground, inset feed, and RF-choke-based biasing network are added to achieve stable tuning and low-loss filtering. The fabricated prototype shows a clear frequency shift with excellent matching and good radiation efficiency. To extend the concept, 2 × 2 and 4 × 4 MIMO filtenna configurations are also developed. Each stage introduces further structural refinement, including inter-element decoupling lines, L-shaped ground extensions, π-shaped shared ground sections, and pairwise high-impedance biasing networks. These features significantly enhance isolation and suppress surface-wave coupling. The proposed MIMO designs provide outstanding diversity performance. An envelope correlation coefficient (ECC) of approximately 10^−2^, a diversity gain close to 10 dB, and a channel capacity loss of less than 0.1 bits/s/Hz are accomplished. Additionally, the antenna exhibits deep total active reflection coefficient (TARC) nulls near − 15 dB, along with mean effective gain (MEG) values that are well-balanced around − 3 dB. Taken as a whole, the results confirm that the developed UWB antenna, its reconfigurable filtenna derivative, and their 2 × 2 and 4 × 4 MIMO extensions form a compact, low-loss, and highly efficient solution for next-generation 5G and cognitive radio systems.

## Introduction

**Fig. 1:**
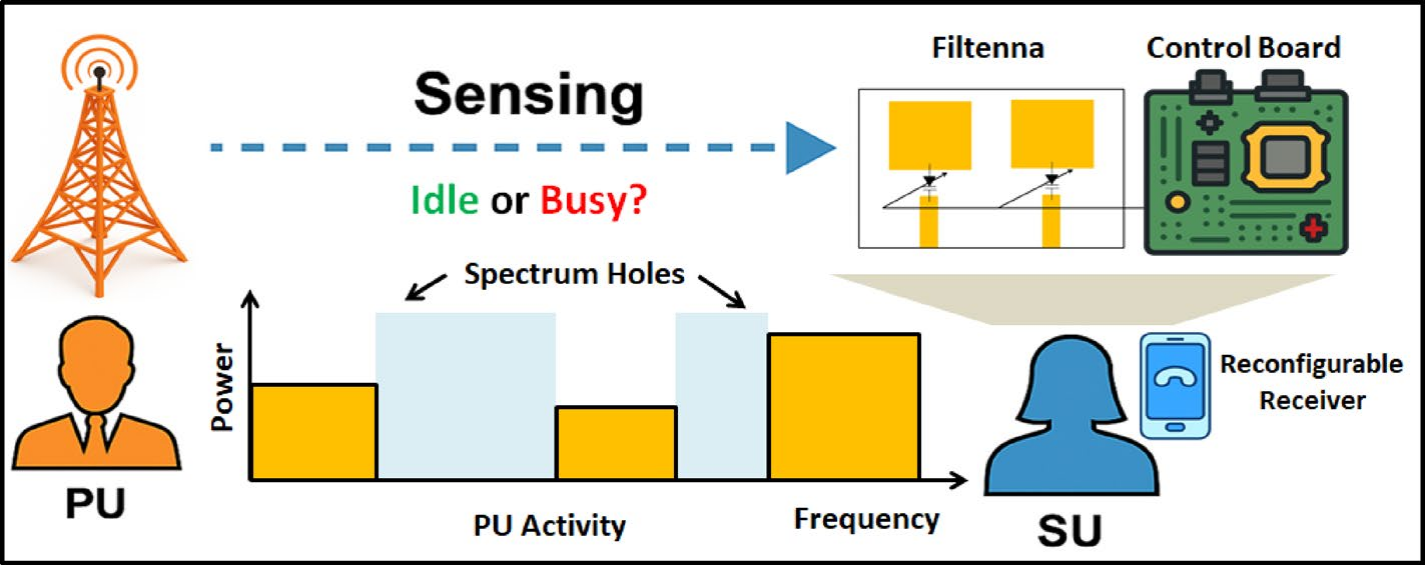
Conceptual diagram of the cognitive radio-based dynamic spectrum access framework.

The rapid evolution of modern wireless communication systems, especially the transition from 4G LTE toward advanced 5G and emerging 6G networks, has created an unprecedented demand for antennas^1,2^. This is due to; they can support high data rates, multi-standard operation, and flexible spectrum utilization. To accommodate the increasing integration density in portable devices, these systems require antennas capable of operating over wide bandwidths. At the same time, they should maintain compact size and efficient radiation characteristics. Conventional narrowband antennas are no longer sufficient, as modern wireless platforms must simultaneously accommodate multiple services. Such services are WLAN, Wi-Fi, WiMAX, sub-6 GHz 5G, and various IoT applications^3^. With the introduction of dynamic spectrum allocation and cognitive radio concepts, the ability of antennas to dynamically reconfigure their operational frequency becomes essential, especially in congested and heterogeneous environments^4–6^. Figure 1 indicates the operating principle of a cognitive radio system. First, the secondary user (SU) performs spectrum sensing (SS) to determine whether the primary user (PU) is active or inactive across different frequency bands. The sensed PU activity reveals occupied regions as well as spectrum holes; those are unused frequency segments that can be safely exploited. Once an idle band is detected, the SU dynamically adjust its operating frequency and access the available channel without causing interference to the PU. This integrated process highlights the core concept of cognitive radio. The intelligent sensing, adaptive filtering, and tunable communication hardware work together, to achieve efficient and interference-aware spectrum utilization. Consequently, frequency agility, compact integration, and high radiation efficiency have become primary design targets in recent antenna engineering research.

Although wideband antennas can cover multiple frequency bands, they often suffer from poor selectivity, strong interference, and reduced radiation efficiency due to their unfiltered nature. Similarly, fixed-band antennas lack the flexibility required to adapt to the continuously changing spectral landscape. This leads to performance degradation in systems where spectrum availability varies over time. Moreover, the integration of multiple antennas on a compact platform introduces severe challenges such as mutual coupling, envelope correlation, and pattern distortion. All of which negatively impact the performance of multiple-input multiple-output (MIMO) systems^7–9^. Many existing reconfigurable antennas either exhibit limited tuning ranges, or provide unstable gain and poor isolation between ports. Therefore, achieving a compact, low-loss, frequency-reconfigurable antenna with stable performance and MIMO compatibility remains an ongoing challenge^10,11^.

Frequency-reconfigurable antennas have emerged as a key enabling technology to address the limitations of conventional narrowband or fixed-band antennas^12–15^. By employing tunable reactive components, most notably varactor and PIN diodes, such antennas can dynamically shift their resonant frequency without altering their physical dimensions. Varactor diodes are particularly attractive for continuous and fine-resolution tuning, as their junction capacitance varies smoothly with the applied reverse-bias voltage^16,17^. This enables accurate frequency agility with minimal power consumption, making them suitable for low-noise and high-efficiency front-ends. In contrast, PIN diodes operate primarily as RF switches, providing discrete ON/OFF states that enable switchable multi-band or mode-reconfigurable behavior, but without the continuous tuning capability offered by varactors^18,19^. Together, these tuning elements allow wireless devices to switch between communication bands. Additionally, they avoid interference, and opportunistically access available spectral holes in cognitive radio systems. Reconfigurable antennas also enhance system flexibility by allowing a single hardware platform to support multiple wireless standards. Thereby, this reduces the need for several dedicated antennas and lowering the overall device footprint. Furthermore, reconfigurability helps conserve power, reduce insertion losses, and improve electromagnetic compatibility in crowded environments. Their ability to maintain stable radiation characteristics over the tuning range is crucial for ensuring consistent link quality in practical applications.

The filtering antenna (filtenna) concept integrates the filter and antenna in a single structure^20,21^. It has recently gained significant attention as an effective approach for improving spectral selectivity while minimizing size, loss, and complexity. Unlike conventional antenna-filter cascades, filtenna eliminates the need for discrete bandpass filters that introduce additional insertion losses and enlarge the hardware footprint. A filtenna inherently shapes the radiation and impedance characteristics of the antenna so that unwanted frequencies are suppressed while the desired band is enhanced. This integrated behavior is particularly beneficial for cognitive radio, and interference-limited environments, where strict control over the operational band is required. Moreover, filtenna provides better impedance matching, improved radiation stability, and reduced parasitic coupling. When combined with frequency reconfigurability, filtenna becomes highly versatile front-end solutions capable of adaptive spectral sensing and communication.

MIMO technology is indispensable in modern wireless networks due to its ability to enhance channel capacity without increasing spectral resources^22,23^. By leveraging spatial diversity, MIMO systems mitigate multipath fading, improve link reliability, and significantly increase data throughput. Such advantages make them essential for applications, such as massive IoT, vehicular communication, and 5G broadband access. However, the performance of MIMO antennas strongly depends on achieving low mutual coupling, low envelope correlation coefficient (ECC), and balanced radiation properties across elements. Poorly designed MIMO antennas suffer from degraded diversity gain, reduced capacity, and higher error rates. For this reason, the development of compact MIMO antennas with efficient isolation techniques, stable gain, and minimal correlation is a major research priority. When frequency reconfigurability is added, MIMO architectures become even more powerful. Because, they able to dynamically adapt to different operating environments and communication standards.

Despite numerous attempts to develop reconfigurable antennas and MIMO structures, significant gaps remain^24^. Many frequency-reconfigurable antennas exhibit narrow tuning ranges or suffer from poor radiation stability as the tuning element varies. Several existing filtenna architectures either lack compactness, require multiple tuning elements that increase complexity, or introduce high insertion losses. Furthermore, only a limited number of studies address the integration of reconfigurability and filtenna behavior within MIMO systems, where mutual coupling and correlation effects become more difficult to control. The majority of reported designs fail to simultaneously achieve wide tunability, high isolation, low ECC, stable gain, and compact integration. In addition, biasing networks often introduce parasitic effects that distort the antenna’s response. This makes practical realization a challenging issue. These limitations highlight the need for a novel MIMO filtenna architecture that provides wide tunability, enhanced selectivity, robust isolation, and stable performance across multiple reconfigurable states.

In this paper, the key contributions can be highlighted as follows:

1. A compact fork-shaped UWB antenna is designed, optimized, fabricated, and experimentally validated, achieving a wide impedance bandwidth from 2.4 to 8 GHz, with stable radiation characteristics. The antenna provides a realized gain reaching approximately 5 dBi at the upper band and a radiation efficiency ranging from 60% at low frequencies to over 90% beyond 5 GHz.
2. A 4 × 4 UWB MIMO configuration is developed based on the proposed element. It provides high isolation, low correlation, and excellent diversity performance across the full band. It maintains a realized gain up to 6 dBi and radiation efficiency exceeding 95% at higher frequencies. In addition, the proposed array demonstrates outstanding MIMO characteristics. It achieves an extremely low envelope correlation coefficient ($ECC\:<\:{10}^{-3}$), near-ideal diversity gain ($DG\:\approx\:\:10\:{\mathrm{dB}}$), minimal channel capacity loss ($CCL\:<\:0.3\:{\mathrm{bits}}/{\mathrm{s}}/{\mathrm{Hz}}$), deep total active reflection coefficient nulls (TARC reaching − 40 dB at resonance), and well-balanced mean effective gain ($MEG\:\approx\:\:-3\:{\mathrm{dB}}$). These results confirm the robustness of the UWB MIMO configuration and its suitability for high-capacity, interference-aware 5G and cognitive radio systems.
3. A frequency-reconfigurable filtenna is introduced by integrating a single varactor-tuned filter with a modified radiating patch, enabling continuous tuning of the operating band from $2.45\:to\:3.48\:{\mathrm{GHz}}$ with low loss and high efficiency. Prototypes of the filtenna are fabricated and tested. The measured design demonstrates a clear tuning shift and close agreement with simulations. It achieves a realized gain between $3.32\:{\mathrm{dB}}i\:\mathrm{a}\mathrm{n}\mathrm{d}\:4.57\:{\mathrm{dB}}i$, and maintaining radiation efficiency between $74.5\:\mathrm{a}\mathrm{n}\mathrm{d}\:81.6\%$ across the tuning states.
4. 2 × 2 and 4 × 4 MIMO filtenna architectures are proposed and evaluated. These designs employ engineered ground structures, decoupling lines, and high-impedance biasing networks. The proposed MIMO architectures reveal significant improvements in isolation, diversity behavior, and overall multiport stability. Through the use of optimized radiating structures, engineered ground modifications, inter-element decoupling features, and high-impedance biasing networks, both configurations effectively suppress mutual coupling and maintain highly consistent performance. The final designs exhibit smooth and well-balanced radiation behavior under simultaneous multiport excitation. Moreover, both MIMO versions maintain uniform power reception and strong resilience against coupling-induced degradation. Together, these results validate the effectiveness and scalability of the proposed architecture in achieving reliable MIMO performance.

The rest of the paper is organized as follows. Section “Proposed fork-shaped circular patch UWB antenna” presents the design evolution and performance analysis of the proposed fork-shaped circular patch UWB antenna. Section “Proposed × MIMO UWB antenna” introduces the 4 × 4 UWB MIMO configuration and discusses its radiation and MIMO characteristics. Section “Proposed frequency reconfigurable filtenna” details the development of the frequency-reconfigurable filtenna and its tuning behavior. Section “Proposed MIMO frequency reconfigurable antenna” extends the concept to 2 × 2 and 4 × 4 MIMO filtenna architectures and evaluates their isolation and diversity performance. Finally, section “Conclusion” concludes the work and highlights the main findings.

## Proposed fork-shaped circular patch UWB antenna

The proposed antenna design undergoes several structural refinements to achieve wideband operation and improved impedance characteristics. A systematic geometric evolution is presented to optimize both the radiator and ground-plane configurations for enhanced UWB performance. In the following subsections, the simulation and fabrication procedures of the proposed antenna are discussed in detail. Also, its performance is evaluated in terms of reflection coefficient, radiation pattern, gain, and efficiency.

### Evolution and design procedure

**Fig. 2:**
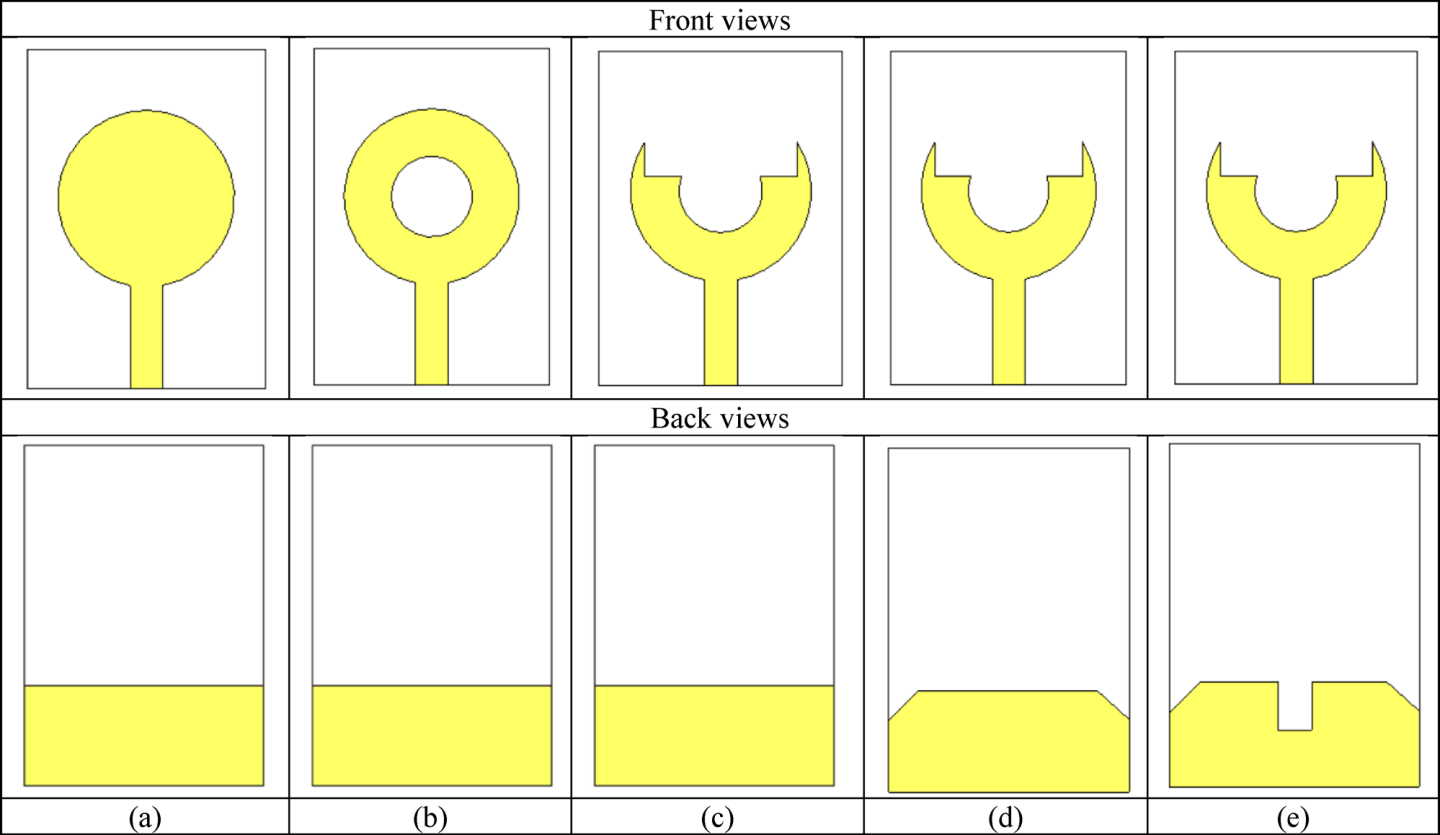
Front and back views of the design evolution of the proposed fork-shaped UWB antenna.

**Fig. 3:**
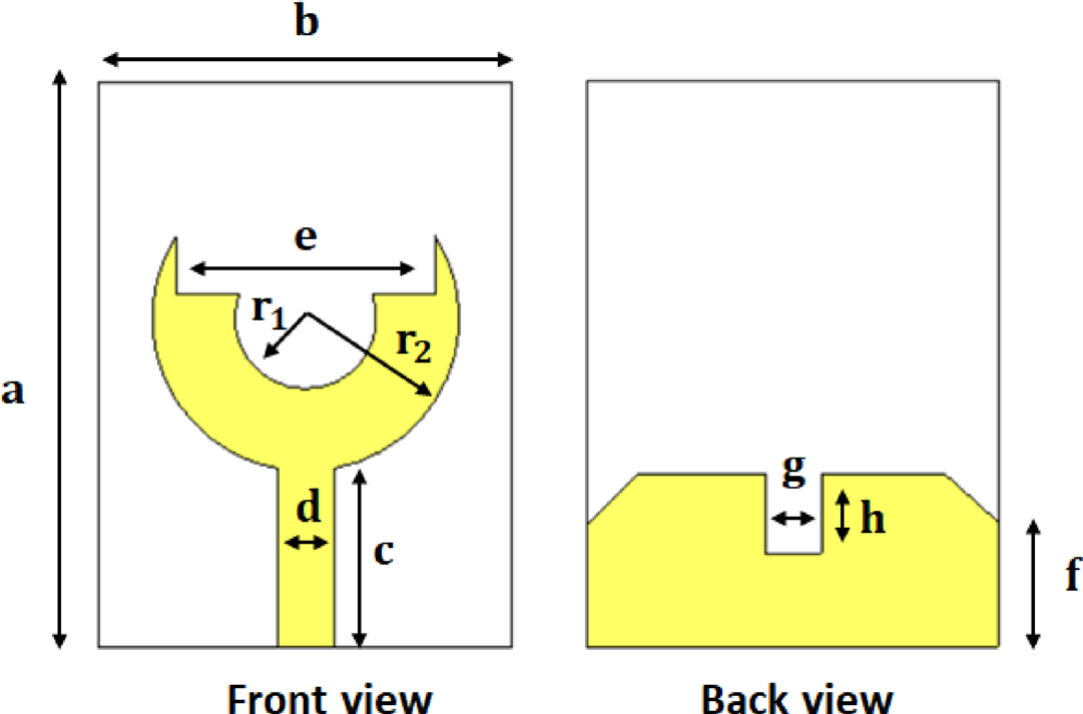
Geometrical parameters of the proposed fork-shaped UWB antenna.

To achieve an optimized geometry of UWB antenna, several design iterations are conducted, as shown in Fig. 2a–e. Initially, a circular patch radiator is adopted owing to its simple structure and stable fundamental resonance. However, it exhibited a narrow bandwidth and poor impedance matching, as reflected in Fig. 2a. To enhance the tuning behavior, a circular slot is introduced at the center of the patch, as shown in Fig. 2b. It increases the current path and introduces an additional resonance. Despite this improvement, the impedance matching at higher frequencies remained insufficient. Subsequently, the radiator is reshaped into a U-shaped, fork-like, configuration, as shown in Fig. 2c. It effectively broadens the surface current distribution and improving the impedance bandwidth. Although fine-tuning of the slot dimensions yields better impedance characteristics at mid-band frequencies, the overall bandwidth was still limited. Finally, a modified ground plane is implemented in the proposed configuration, as shown in Fig. 2d. The partial ground with a central notch enables better coupling between the radiator and ground, as shown in Fig. 2e. This adjustment results in a significant enhancement of the operating bandwidth and a deeper resonance. The proposed design achieved a much wider operational bandwidth covering both the 3.5 GHz and 6 GHz bands, confirming its suitability for 5G applications. The proposed antenna geometry with its front and back views is illustrated in Fig. 3. The corresponding geometrical parameters are summarized in Table 1, which provides the detailed values of each parameter used in the antenna optimization and fabrication process.

**Fig. 4:**
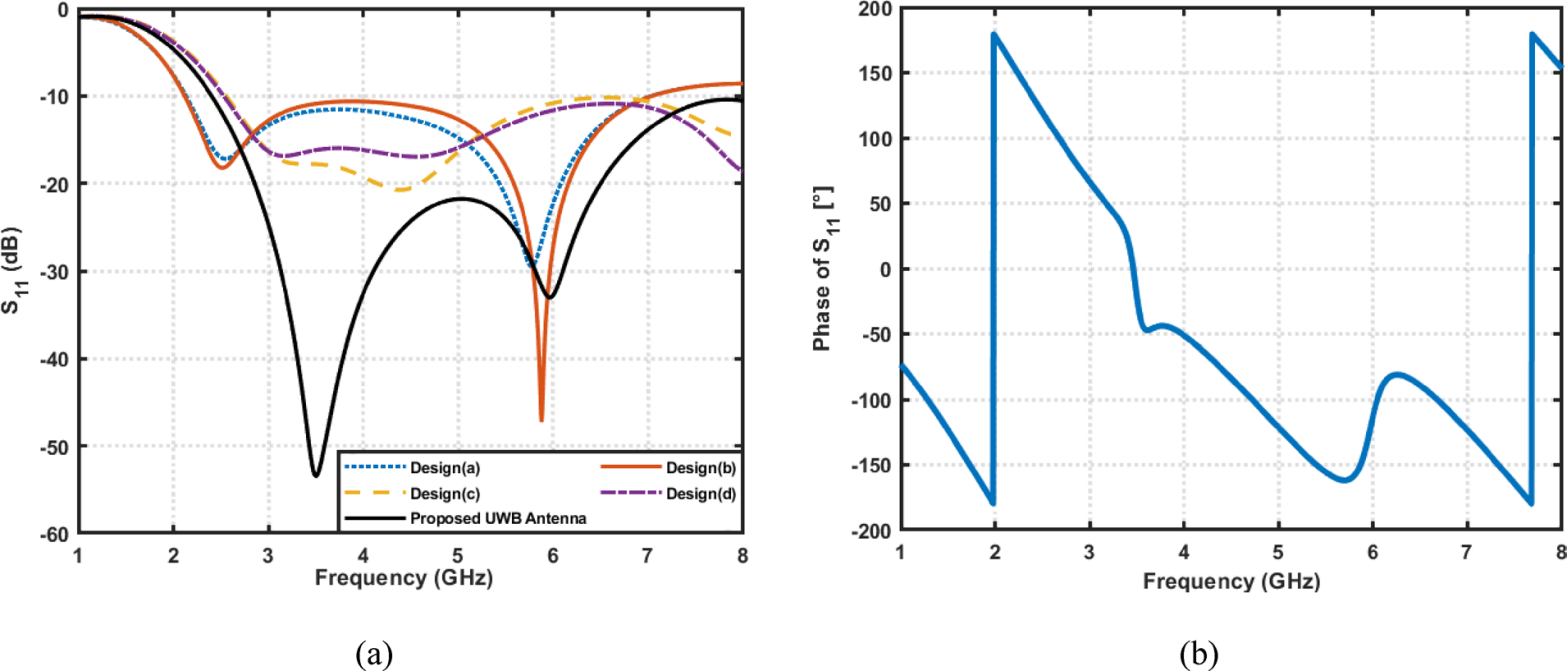
(**a**) Magnitude of simulated reflection coefficient $\left|{S}_{11}\right|$​ for different design iterations. (**b**) Phase of simulated reflection coefficient $\angle\:{S}_{11}$ for the proposed UWB antenna.

The design and simulation of the proposed antenna are performed using computer simulation technology (CST) Microwave Studio. Figure 4a shows the simulated reflection coefficients $\left|{S}_{11}\right|$ for the different design stages. It is observed that the proposed fork-shaped circular patch antenna achieves a remarkable improvement in impedance matching and bandwidth compared to the earlier prototypes. The final antenna exhibits two dominant resonances around $3.5\:{\mathrm{GHz}}\:\mathrm{a}\mathrm{n}\mathrm{d}\:6\:{\mathrm{GHz}}$, providing an overall impedance bandwidth from $2.4\:\mathrm{t}\mathrm{o}\:8\:{\mathrm{GHz}}$ for $\left|{S}_{11}\right|\:\:\le\:\:-10\:{\mathrm{dB}}$. This exceptionally wide bandwidth ($5.6\:{\mathrm{GHz}}$) classifies the antenna as Ultra-Wideband (UWB). Such a broadband response enables the antenna to support a variety of wireless applications, including the 2.4 GHz ISM band, Wi-Fi (2.4/5/6 GHz), WiMAX (3.5 GHz), 5G sub-6 GHz communication. Also, Fig. 4b illustrates the reflection phase of the proposed UWB antenna over the wide operating band from $2.4\:\mathrm{t}\mathrm{o}\:8\:\mathrm{G}\mathrm{H}\mathrm{z}$. The phase response exhibits a smooth and continuous variation across the band, which is characteristic of wideband radiating elements without inherent filtering behavior.

**Fig. 5:**
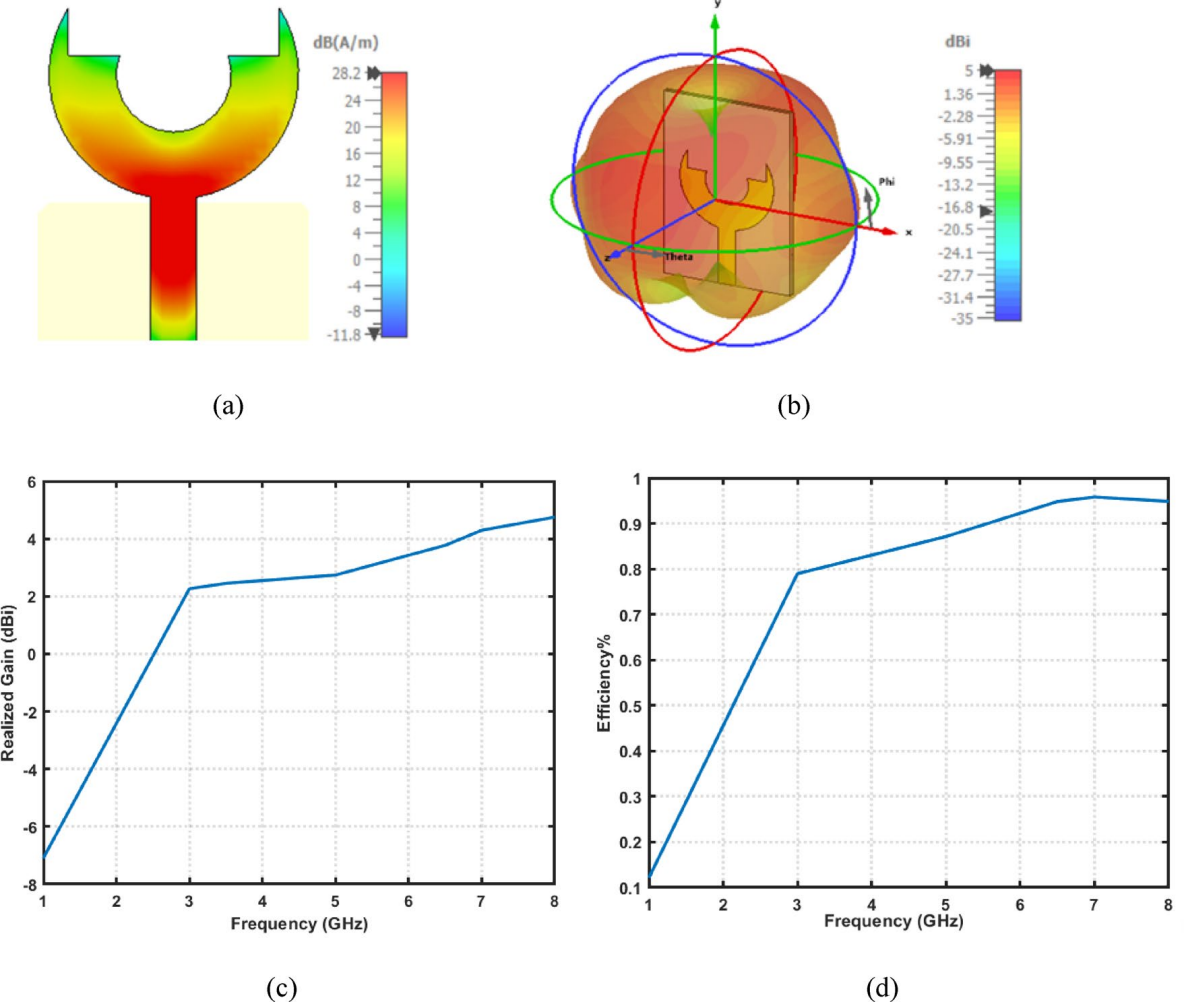
Electromagnetic characteristics of the proposed UWB antenna (**a**) surface current distribution at $f=8\:{\mathrm{GH}}$, (**b**) 3D radiation pattern, (**c**) Realized gain versus frequency, and (**d**) radiation efficiency versus frequency.

Figure 5a shows the surface current distribution of the proposed fork-shaped circular antenna. It is evident that the maximum current density is concentrated along the feed line and the lower region of the circular patch. This indicates that these areas play a dominant role in radiation and impedance matching. The smooth current transition over the patch confirms the effective excitation of the fundamental mode and stable wideband operation. Also, the 3D radiation pattern of the proposed antenna is illustrated in Fig. 5b. The antenna exhibits an almost omnidirectional radiation behavior in the H-plane and a dipole-like pattern in the E-plane. This uniform radiation makes the antenna suitable for mobile and cognitive radio environments, where multi-directional reception is essential. Additionally, the simulated realized gain versus frequency is presented in Fig. 5c. It is found that the antenna maximum gain is about $5\:{\mathrm{dB}}i$ at $8\:{\mathrm{GHz}}$. The gain stability within the operational band highlights the effectiveness of the proposed geometry in maintaining efficient radiation across the ultra-wideband range. Furthermore, Fig. 5d illustrates the simulated radiation efficiency of the proposed antenna. The efficiency rises rapidly from around $60\%$ at 2.4$\:{\mathrm{GHz}}$ to above $90\%$ beyond$\:5\:{\mathrm{GHz}}$. Such high efficiency confirms the low loss characteristics of the antenna, which can be attributed to the optimized geometry and proper impedance matching.

**Table 1:** Optimized design parameters of the proposed antenna geometry.

Parameters	Dimensions (mm)	Parameters	Dimensions (mm)
$\mathrm{a}$	$50$	$\mathrm{f}$	10.5
$\mathrm{b}$	*35*	$\mathrm{g}$	4.7
$\mathrm{c}$	15	$\mathrm{h}$	7
$\mathrm{d}$	4.7	${\mathrm{r}}_{1}$	6
$\mathrm{e}$	*22*	${\mathrm{r}}_{2}$	*13*

### Fabrication and measurement setup

**Fig. 6:**
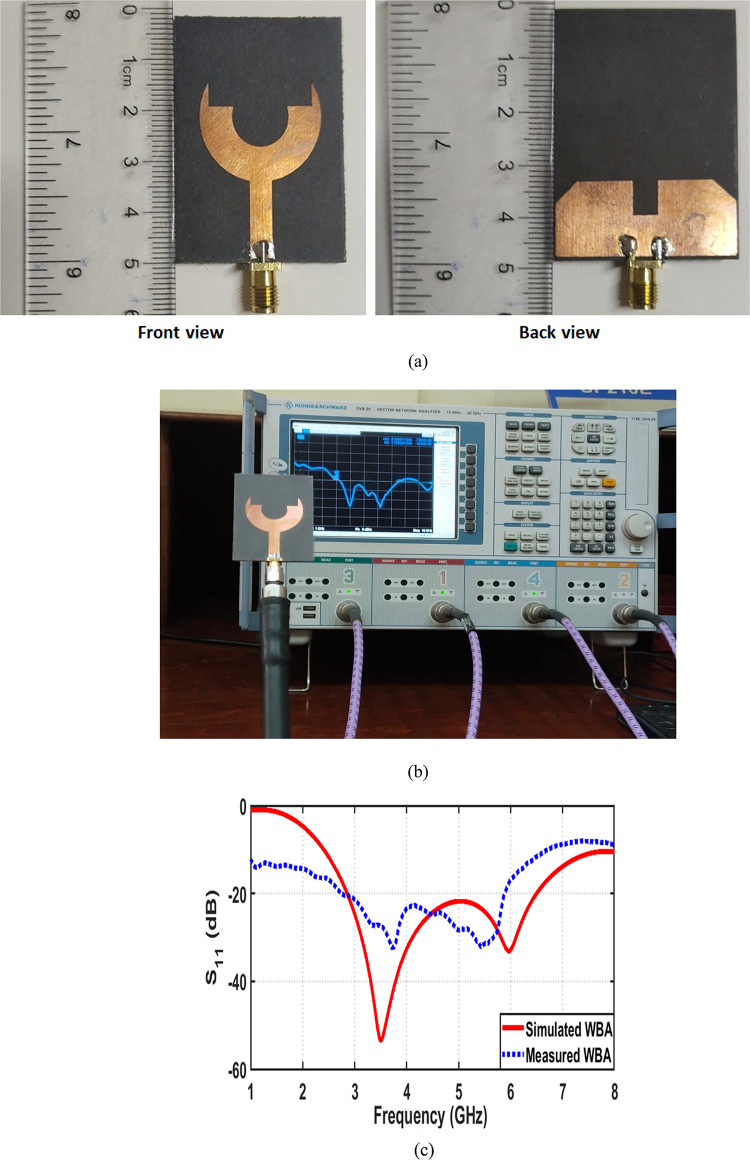
Fabrication and experimental validation of the proposed UWB antenna. (**a**) Fabricated prototype of the proposed antenna with actual physical dimensions, (**b**) measurement setup, and (**c**) comparison between the simulated and measured reflection coefficient of the proposed UWB antenna.

**Fig. 7:**
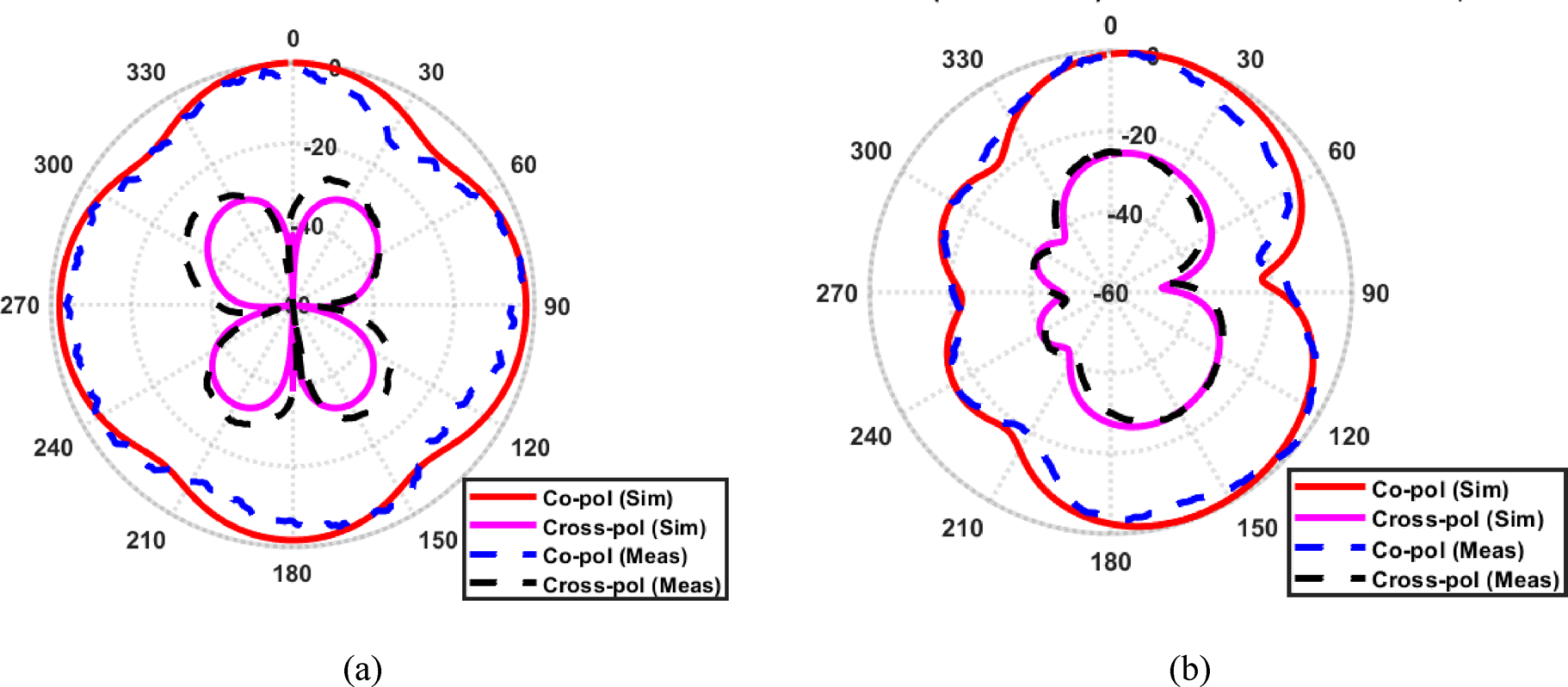
Normalized co-polarized and cross-polarized radiation patterns of the proposed UWB antenna, showing simulated and measured results in the principal planes. (**a**) at ${\upphi\:}=0^\circ$ and (**b**) at $\phi\:=90^\circ$.

The fabricated prototype of the proposed fork-shaped circular patch antenna is shown in Fig. 6a. The antenna is fabricated using standard photolithographic etching on a Rogers 5880 substrate with a thickness of$\:1.57\:{\mathrm{mm}}$, dielectric constant of $2.2$, and low loss tangent of 0.0009. The reflection coefficient of the fabricated antenna IS experimentally measured using a Vector Network Analyzer (VNA), model R&S ZVB20 from Rohde & Schwarz, covering the frequency range of 10 MHz to 20 GHz. The measurement setup is shown in Fig. 6b, where the fabricated antenna is connected to the VNA through a standard SMA connector and high-quality coaxial cable to minimize measurement losses. Prior to measurement, the VNA was calibrated using a standard Short–Open–Load–Through (SOLT) calibration to ensure high accuracy. The measured $\left|{S}_{11}\right|$ result is then compared with the simulated one, showing good agreement as in Fig. 6c. It is worthy mentioned that the measured reflection coefficient generally follows the simulated response, with a slight frequency shift and reduced depth of the resonant notches. These minor discrepancies can be attributed to substrate parameter variations, fabrication inaccuracies, connector and soldering losses, as well as measurement setup uncertainties such as, cable effects and calibration limitations. Such variations are commonly reported in practical UWB antenna measurements and do not affect the overall impedance bandwidth or the antenna’s operational performance. The radiation characteristics of the proposed antenna are experimentally evaluated in an anechoic chamber using a standard far-field measurement setup. The antenna under test (AUT) was connected to a vector network analyzer and mounted on a rotating positioner, while a linearly polarized horn antenna was used as the transmitting antenna. For co-polarization measurements, the polarization of the transmitting horn is aligned with that of the AUT. For cross-polarization measurements, the transmitting antenna is rotated by 90°, while keeping the AUT orientation unchanged. The antenna was rotated over 360° to record the radiation patterns in the principal planes. Figure 7 presents the simulated and measured normalized co- and cross-polarized radiation patterns of the proposed fork-shaped circular antenna at $\phi\:\:=\:0^\circ$ and $\phi\:\:=\:90^\circ$ planes. A good agreement between the measured and simulated results is observed for both polarization components. Minor discrepancies between simulation and measurement are mainly attributed to fabrication tolerances, connector losses, and measurement setup imperfections. As observed, the co-polarized radiation component dominates the radiation behavior of the proposed antenna, defining a clear and stable main beam. In contrast, the cross-polarized component is significantly suppressed over the entire angular range, particularly within the main beam region, where it remains more than $20\:{\mathrm{dB}}$ lower than the co-polarized component.

## Proposed × MIMO UWB antenna

Multiple-Input Multiple-Output antenna systems play a crucial role in modern wireless communication technologies, particularly in 5G and beyond networks. By employing multiple antennas at both the transmitter and receiver, MIMO systems significantly enhance channel capacity, data throughput, and link reliability. These are accomplished without requiring additional bandwidth or transmission power. Moreover, the use of diversity and spatial multiplexing techniques helps mitigate multipath fading. They improve signal quality in complex propagation environments. In addition, MIMO antennas enable efficient spectrum utilization and support advanced technologies such as massive MIMO, beamforming, and spatial diversity. These technologies are fundamental for achieving high-speed and low-latency communication. Therefore, designing compact, wideband, and high-isolation MIMO antenna structures has become essential for next-generation wireless systems.

### Design process and radiation characteristics

**Fig. 8:**
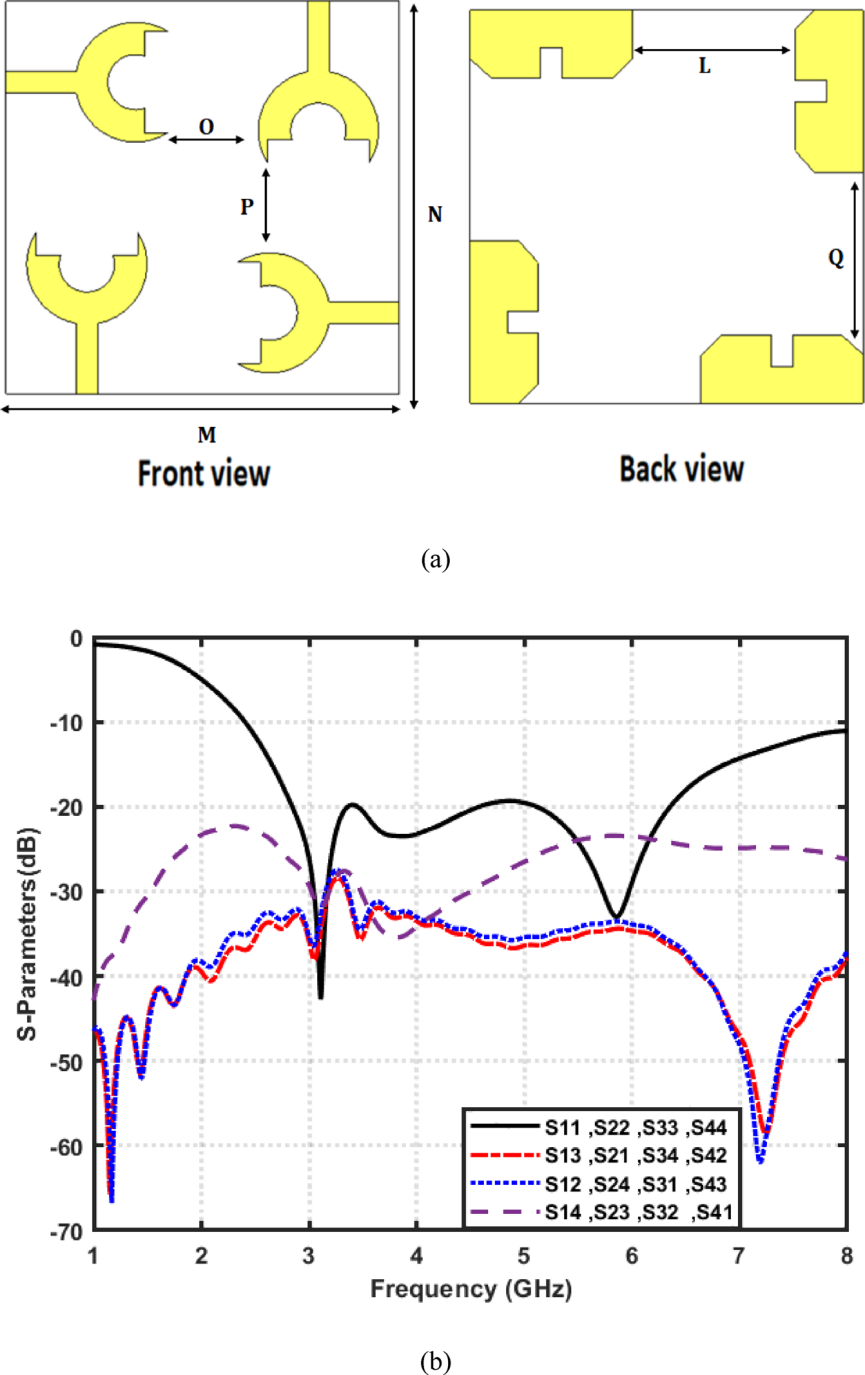
Proposed 4 × 4 MIMO UWB antenna configuration and its simulated S-parameters. (**a**) Front and back views of the proposed 4 × 4 MIMO antenna layout and (**b**) Simulated reflection and transmission coefficients versus frequency.

**Fig. 9:**
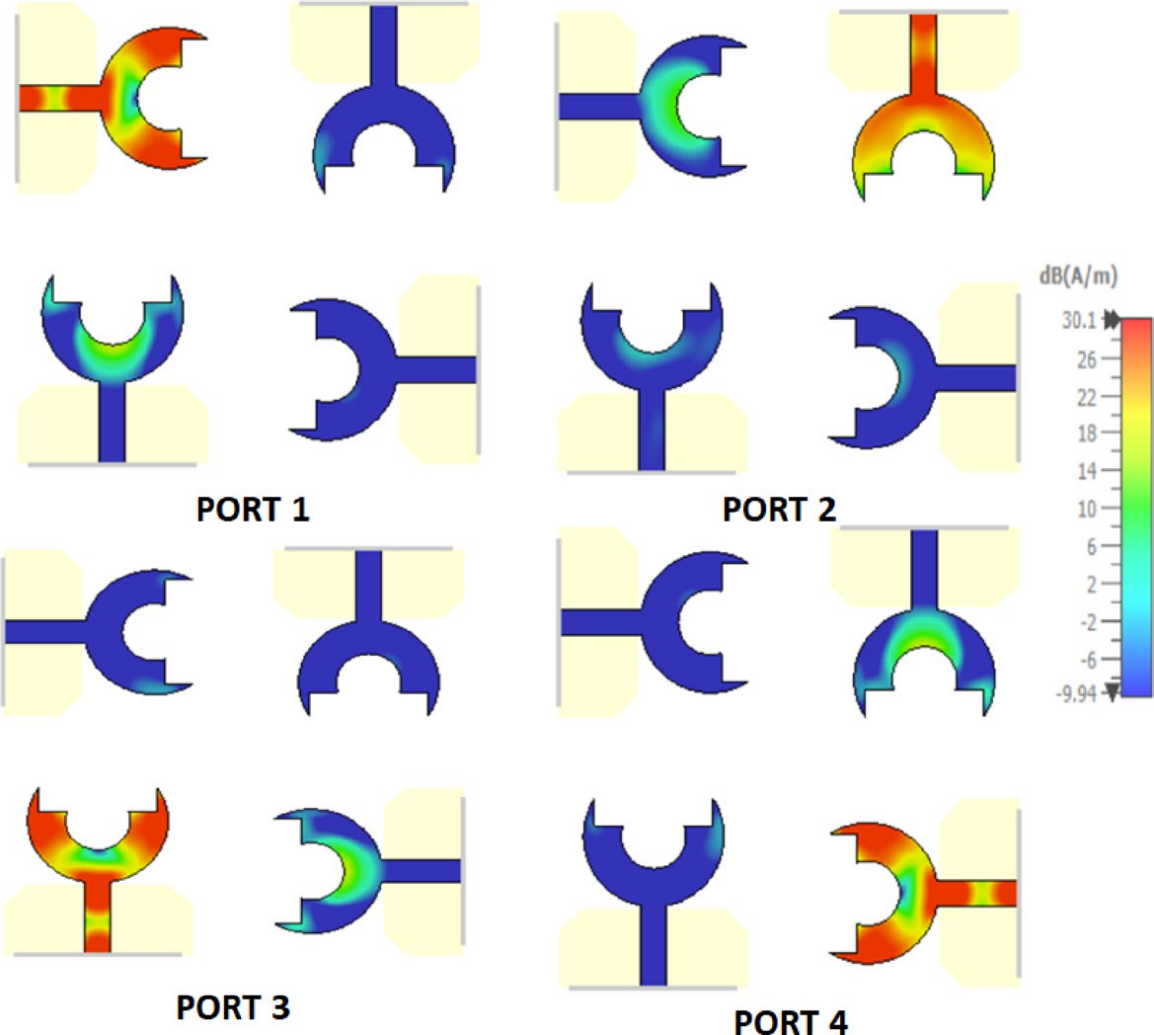
Surface current distribution of the proposed 4 × 4 MIMO antenna at the four excitation ports.

**Fig. 10:**
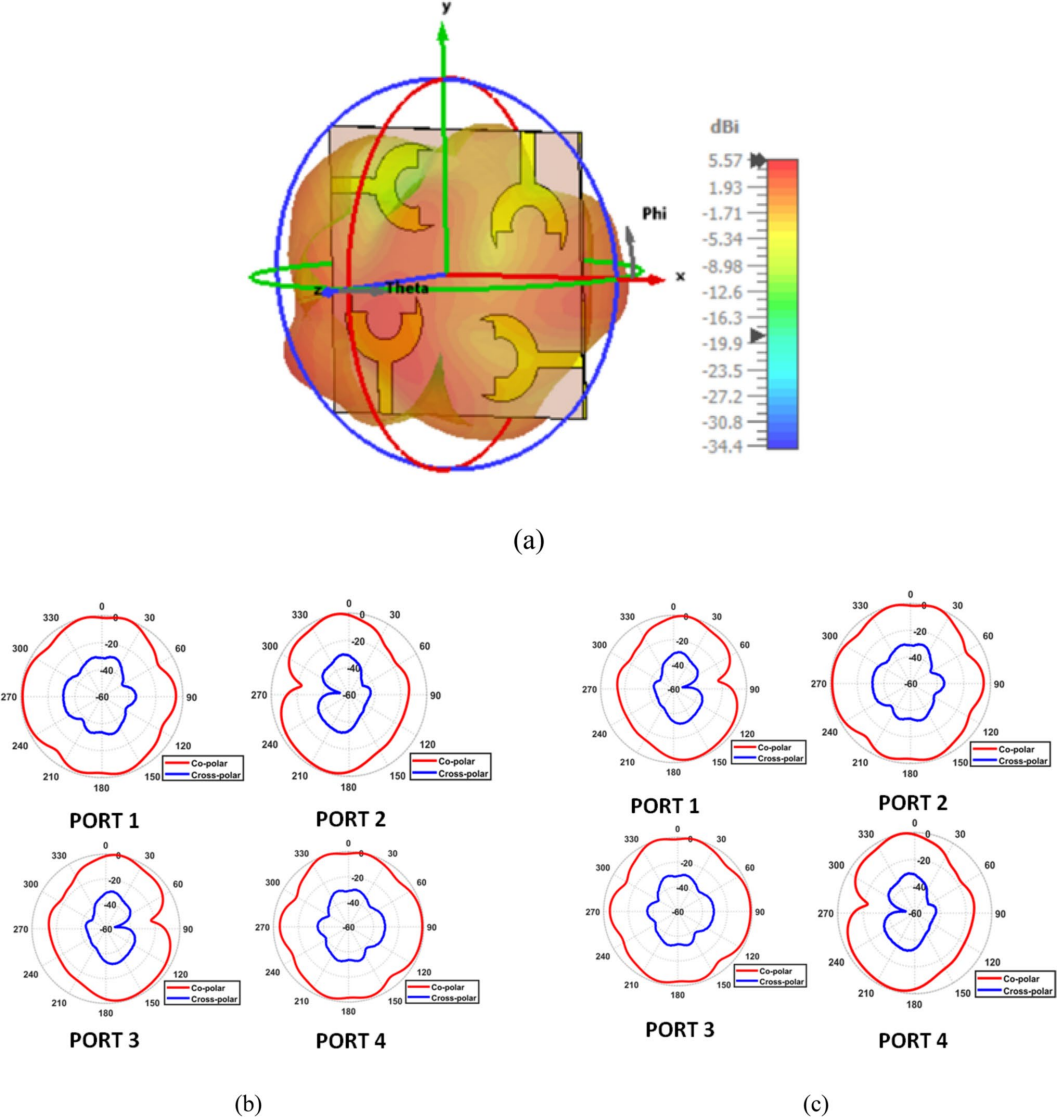
Radiation performance of the proposed 4 × 4 MIMO antenna. (**a**) 3D radiation pattern, (**b**) simulated normalized co- and cross-polarized 2D radiation patterns for individual port excitations at $\phi\:=0^\circ$, and (**c**) simulated normalized co- and cross-polarized 2D polar radiation patterns for individual port excitations at $\phi\:=90^\circ$.

**Fig. 11:**
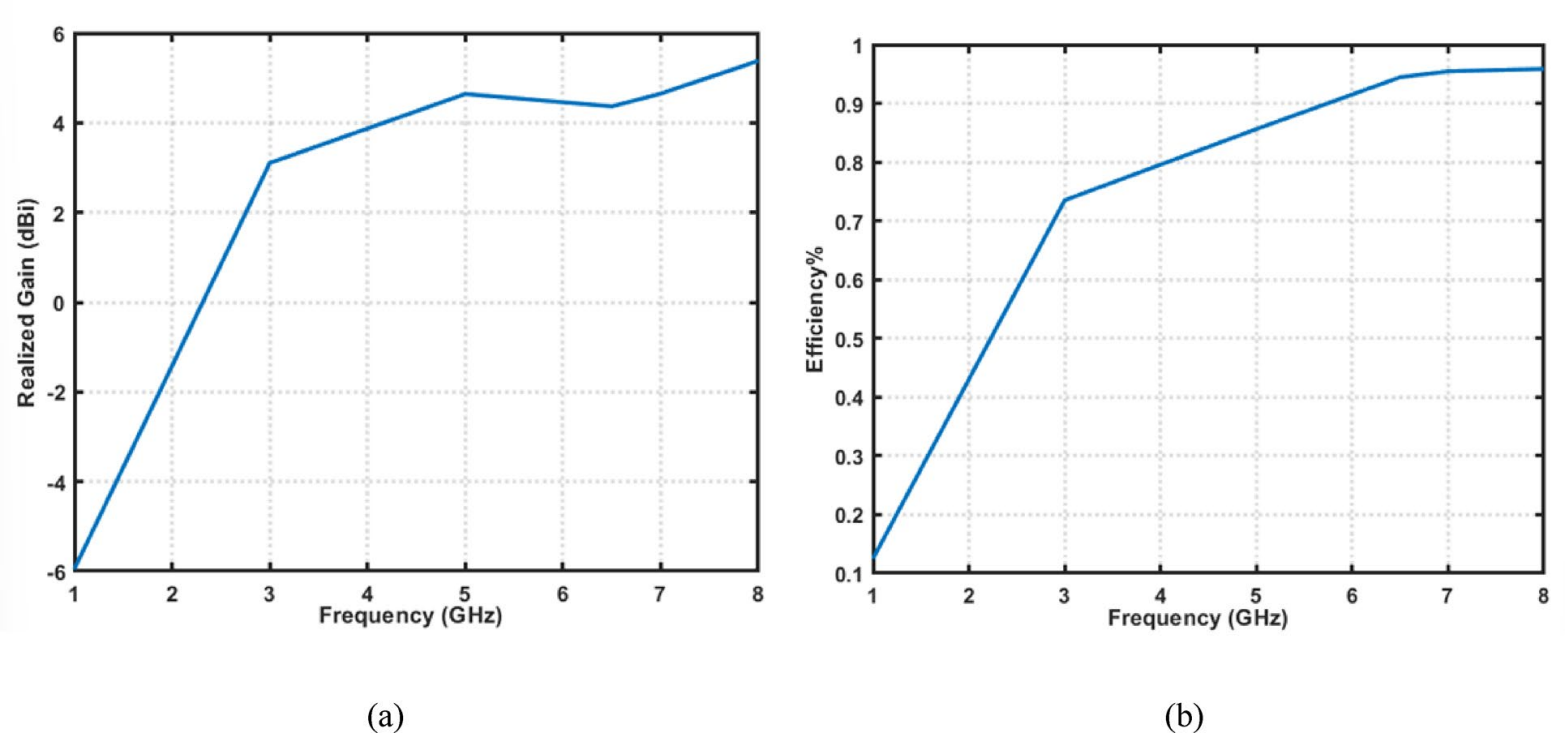
Performance characteristics of the proposed 4 × 4 MIMO antenna. (**a**) Realized gain and (**b**) radiation efficiency versus frequency.

Front view and back view of the proposed 4-port MIMO antenna are shown in Fig. 8a. It is developed by arranging four identical fork-shaped circular radiating elements orthogonally at the four corners of the substrate. Each element is excited through an independent microstrip feed line, while maintaining a symmetric configuration to minimize mutual coupling and achieve pattern diversity. All geometrical dimensions of the proposed structure are specified in millimeters, where M = 85, *N* = 85, O = 20, L = 35, *P* = 21.5, and Q = 39.5. Figure 8b presents the simulated S-parameters of the proposed 4-port MIMO antenna configuration. The parameters $\left|{S}_{11}\right|,\:\left|{S}_{22}\right|$, $\left|{S}_{33}\right|$, and $\left|{S}_{44}\right|$ represent the return losses of the four antenna elements. They exhibit good impedance matching across a wide frequency range from $2.4\:\mathrm{t}\mathrm{o}\:8\:{\mathrm{GHz}}$. This confirms the UWB behavior of each element. The mutual coupling between the antenna ports is indicated by the transmission coefficients$\:({S}_{12},\:{S}_{13},\:{S}_{14},\:{S}_{21},\dots\:\mathrm{e}\mathrm{t}\mathrm{c})$. It is observed that the isolation between adjacent and diagonal elements remains better than $23\:{\mathrm{dB}}$ throughout the operational band. Such low coupling levels demonstrate the effectiveness of the orthogonal placement in suppressing surface-current interaction between elements. Figure 9 shows the simulated surface current distributions of the proposed 4-port MIMO antenna. Each port is excited individually, while the remaining ports are terminated with a 50 Ω load. It can be clearly observed that the strong current concentration is localized around the excited radiating element and its adjacent feed line. On the other hand, the non-excited elements exhibit very weak induced currents. Moreover, the current paths follow the fork-shaped circular patch contour, validating that the main radiation mechanism is dominated by the edges of the fork aperture. The minimal current interaction between the adjacent and diagonal elements further proves that the antenna configuration effectively suppresses surface-wave propagation and near-field coupling. Figure 10a shows the 3D radiation pattern of the proposed 4-port MIMO antenna. Also, Fig. 10b and c present the simulated normalized co- and cross-polarized 2D polar radiation patterns of the proposed 4 × 4 UWB MIMO antenna for individual port excitations in the E-plane $(\phi\:\:=\:0^\circ\:)$ and H-plane$\:(\phi\:\:=\:90^\circ\:)$, respectively. The patterns demonstrate consistent radiation behavior across all ports, with the cross-polarized component remaining approximately $25\:{\mathrm{dB}}$ lower than the co-polarized component within the main beam region, indicating good polarization purity. As shown in Fig. 11a, the realized gain increases steadily with frequency, until reaching up to about $6\:{\mathrm{dB}}i$ at 8 GHz. Furthermore, Fig. 11b demonstrates that the antenna maintains high radiation efficiency, beginning around $60\%$ at the lower band and rising to over 95% at higher frequencies.

**Fig. 12:**
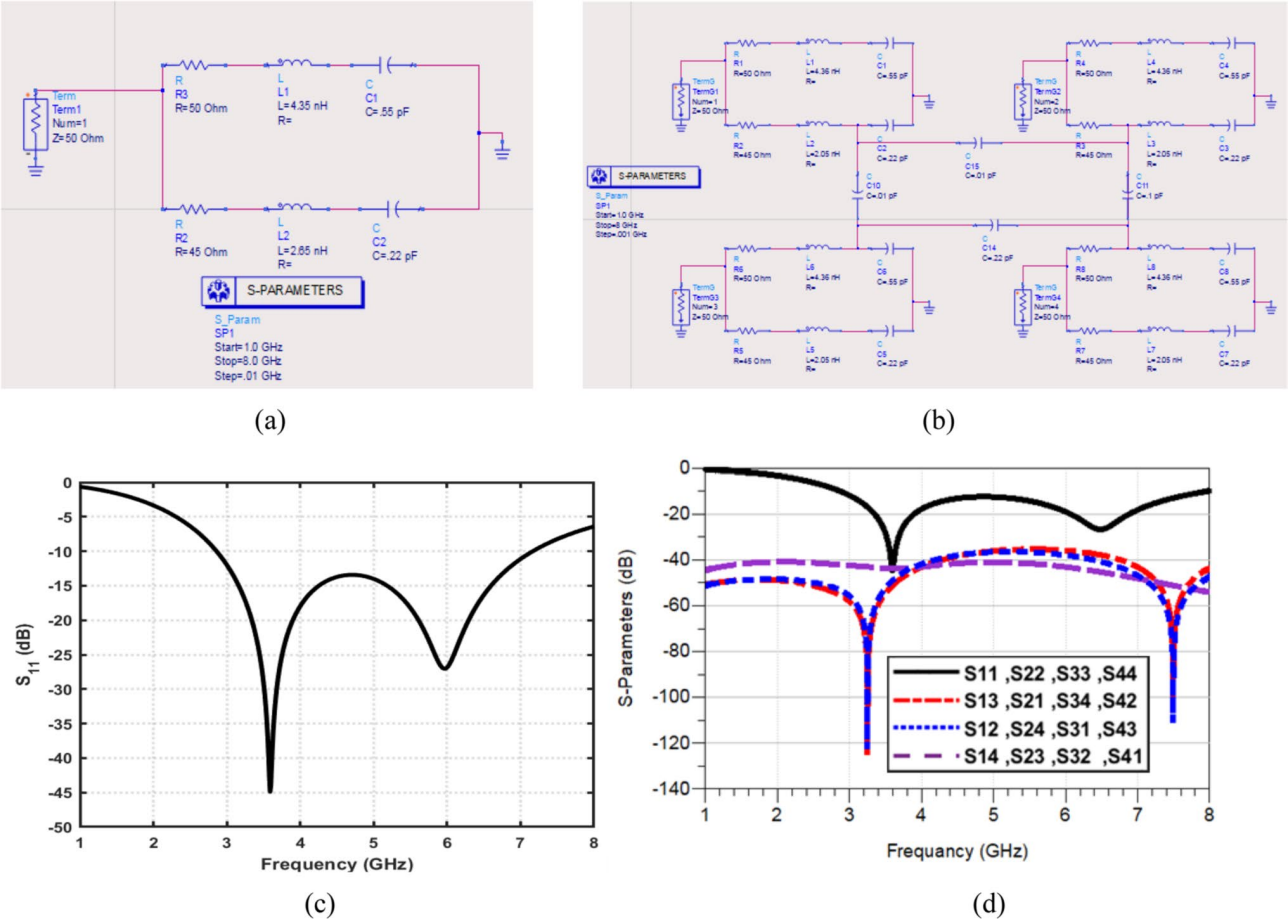
ADS-based equivalent circuit modeling and validation of the proposed antenna configurations. (**a**) Equivalent circuit model of the proposed single UWB antenna. (**b**). Simulated $\left|{\mathrm{S}}_{11}\right|$of the single-antenna equivalent circuit. (**c**) Equivalent circuit model of the proposed 4 × 4 MIMO antenna. (**d**) Comparison of S-parameters obtained from the equivalent circuit model of the 4 × 4 MIMO antenna, demonstrating accurate prediction of impedance matching and inter-port isolation.

Figure 12a presents the extracted equivalent circuit model of the proposed single UWB antenna, implemented and simulated using the Keysight advanced design system (ADS). The radiating patch is represented by a cascaded RLC resonant network that accurately captures the fundamental and higher-order resonances observed in the full-wave CST simulations. The series inductances and capacitances model the effective current paths along the fork-shaped radiator, while the resistive elements account for conductor and dielectric losses. In addition, Fig. 12b illustrates the equivalent circuit representation of the proposed 4 × 4 MIMO antenna configuration. Each antenna element is modeled using the same validated single-element RLC network, while the mutual coupling between antenna ports is represented through additional coupling branches connecting the individual resonant circuits. These interconnection paths effectively model the electromagnetic interaction between adjacent and diagonal elements, including surface-wave coupling and near-field interactions. The simulated S-parameters of proposed single UWB antenna and the proposed 4 × 4 MIMO antenna configuration obtained from the ADS-based equivalent circuit models are presented in Fig. 12c, d, respectively. The strong correspondence between the ADS circuit-based and full-wave CST results demonstrates that the proposed equivalent circuit models provide a reliable and physically meaningful interpretation of the antenna behavior. Moreover, it offers valuable insight into the role of the inter-element coupling paths and impedance interactions in achieving enhanced isolation in the 4-port MIMO configuration.

### MIMO evaluation parameters

To comprehensively evaluate the performance and diversity characteristics of a MIMO antenna system, several key parameters must be considered as follows^25–28^.

#### Envelope correlation coefficient

The Envelope Correlation Coefficient (ECC) quantifies the correlation between antenna elements, where a low value, typically$\:<\:0.5$, indicates good diversity performance and minimal signal interference.

#### Diversity gain

The Diversity Gain (DG) represents the improvement in received signal quality provided by multiple antennas compared to a single-antenna system. Although diversity gain of $10\:{\mathrm{dB}}$ is considered the theoretical optimum in realistic propagation environments, the proposed antenna exhibits a DG exceeding $9.999\:{\mathrm{dB}}$ between adjacent elements, indicating excellent diversity. This result confirms that the application of diversity techniques can significantly enhance the system’s reliability and throughput.

#### Total active reflection coefficient

The Total Active Reflection Coefficient (TARC) assesses the combined reflection behavior of all active ports. This ensures that mutual coupling does not significantly degrade the overall impedance matching.

#### Mean effective gain

The Mean Effective Gain (MEG) estimates the average received power from each antenna element under random signal conditions. It characterizes the antenna’s performance in non-line-of-sight (NLOS) environments. It represents the average power received by each antenna element under multipath conditions. An MEG value of $-3\:{\mathrm{dB}}$ corresponds to $100\%$ effective gain efficiency. For optimal diversity performance, all MIMO elements should exhibit approximately equal MEG values. Since MEG inherently accounts for mutual coupling between antenna elements, a lower MEG value implies better system sensitivity. However, it may introduce higher design complexity or cost.

#### Channel capacity loss

**Fig. 13:**
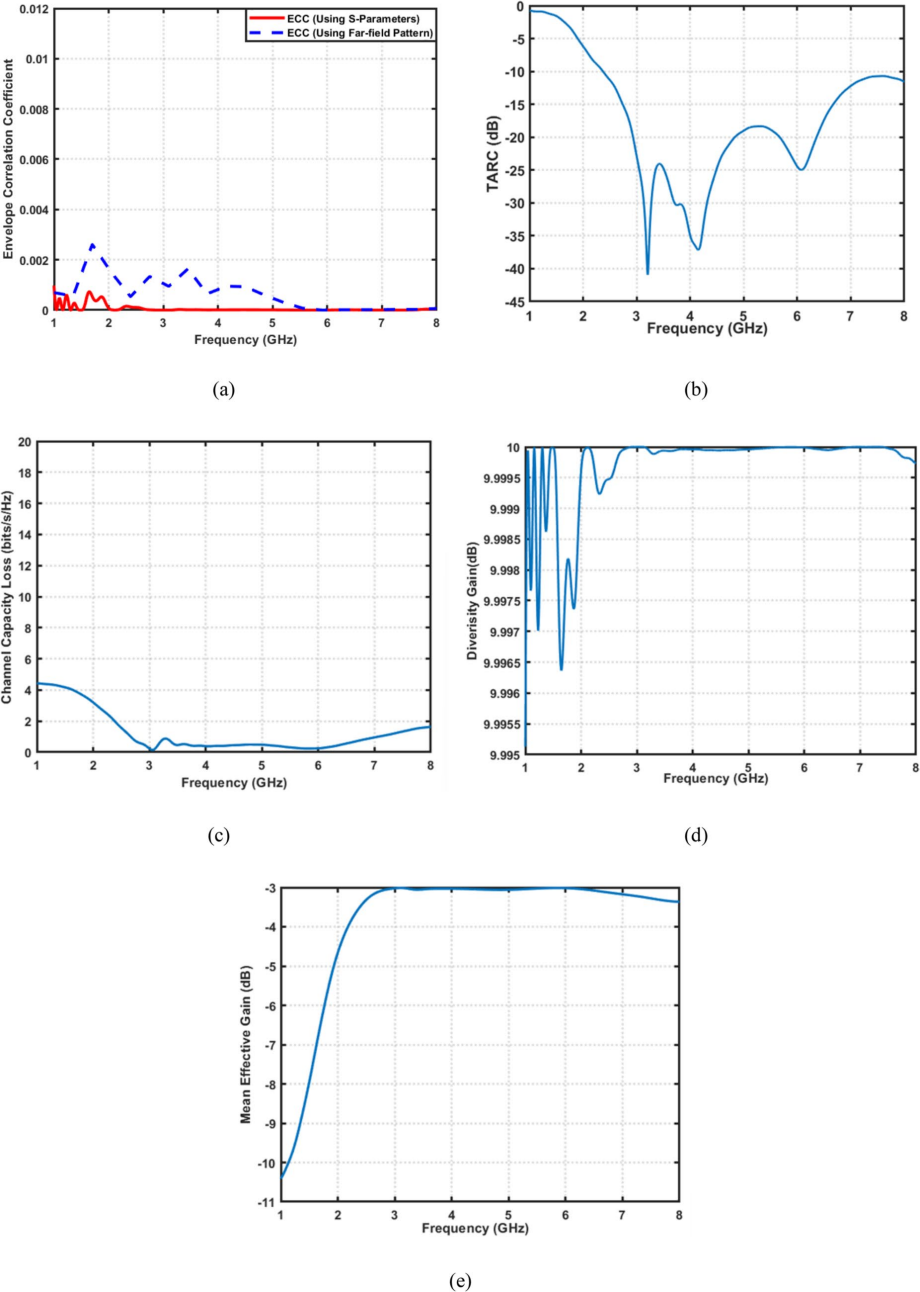
MIMO performance analysis of the proposed 4 × 4 MIMO antenna. (**a**) ECC, (**b**) TARC, (**c**) CCL, (**d**) DG, and (**e**) MEG versus frequency.

The Channel Capacity Loss (CCL) evaluates how much information capacity is lost due to correlation and coupling, directly reflecting the system’s ability to achieve high data throughput. It quantifies the reduction in data capacity caused by correlation and coupling among MIMO antenna elements. It is one of the most critical parameters for assessing the information throughput capability of a MIMO system. A low CCL value $< ~0.3~\;{\mathrm{bits/s/Hz}}$) signifies minimal correlation and high channel capacity efficiency. Figure 13a compares the envelope correlation coefficient extracted using the conventional S-parameter-based formulation and the more rigorous radiation-pattern-based method for the proposed UWB MIMO antenna. As observed, both approaches yield consistently low ECC values across the entire ultra-wideband frequency range (2.4–8 Ghz), remaining well below 0.01. The radiation-based ECC exhibits slightly higher values than the S-parameter-based counterpart, which is expected due to its direct dependence on the far-field radiation characteristics rather than port coupling alone. Nevertheless, the close agreement between the two methods confirms that the proposed antenna maintains excellent spatial diversity and highly uncorrelated radiation patterns throughout the wide operating bandwidth. Figure 13b presents the TARC results. The TARC remains lower than $-10\:{\mathrm{dB}}$ across most of the operating band, with deep nulls reaching around $-40\:{\mathrm{dB}}$ near $3\:{\mathrm{GHz}}$ and $5.8\:{\mathrm{GHz}}$. These low TARC levels confirm excellent impedance matching and low reflection losses under simultaneous multiport excitation. Hence, the antenna maintains stable active performance and minimal mutual coupling. Figure 13c shows the CCL as a function of frequency. The CCL remains below $0.3\:bits/s/Hz$ across the entire range, achieving a minimum value close to zero near $3\:{\mathrm{GHz}}$. This result confirms that the proposed antenna causes negligible channel capacity degradation. This implies high spectral efficiency and improved data throughput for MIMO communication systems. Figure 13d depicts the DG versus frequency. The DG remains nearly constant and close to $10\:{\mathrm{dB}}$ across the full band, indicating excellent diversity performance. This validates that the antenna can effectively mitigate multipath fading, guaranteeing reliable signal reception in rich scattering environments. Finally, Fig. 13e illustrates the MEG for both antenna elements. The MEG values are approximately $-3\:{\mathrm{dB}}$ over the entire frequency band, which is the ideal theoretical value for balanced MIMO antennas.

### Comparison with related work

A comprehensive comparison between the proposed 4-port frequency-reconfigurable MIMO filtenna and several recently published multi-port MIMO antenna systems is presented as in Table 2. In summary, the proposed work demonstrates highly competitive performance relative to the existing literature. Also, it achieves a highly balanced combination of wide tunability, strong radiation performance, superior isolation, and excellent MIMO diversity metrics. This highlights the novelty and practical strength of the proposed design within next-generation wireless systems. Although higher-order MIMO antennas have been reported, a four-port configuration offers a favorable balance between diversity performance, system complexity, and practical implementation. For UWB and cognitive radio applications, the proposed 4-port design provides sufficient spatial diversity and low correlation while maintaining compact size and manageable fabrication complexity.

**Table 2:** Performance comparison of recently reported 4-port UWB MIMO antennas.

Reference	Antenna size (mm)	Number of ports	Frequency (GHz)	Peak efficiency (%)	Gain (dBi)	Isolation (dB)	ECC	MEG (dB)	CCL (bit/s/Hz)	DG (dB)	TARC (dB)
^29^	90 × 90	4	2.7–12	80	5	<− 15	< 0.1	− 3	N/A	$>9.97$	N/A
^30^	60 × 60	4	3–11	68	3.4	<− 20	< 0.02	− 3	< 0.4	> 9.98	<− 10
^31^	58 × 58	4	2.8–12.1	N/A	5.96	<− 20	> 0.03	− 3	< 0.4	> 9.99	<− 10
^32^	40 × 40	4	3.3–13.7	89	5.5	<− 18	< 0.012	− 3.1	< 0.3	> 9.998	N/A
^33^	44 × 44	4	3.2–12.4	89	4.9	<− 26	< 0.0016	− 3.1	< 0.31	> 9.96	N/A
^34^	45 × 45	4	3.3–13.1	73	4	<− 17	< 0.02	N/A	N/A	> 9.998	<− 25
Proposed work	85 × 85	4	2.4–8	97	5.5	<− 23	< 0.001	− 3	< 0.3	> 9.999	<− 10

## Proposed frequency reconfigurable filtenna

A frequency-reconfigurable filtenna integrates filtering and radiation functions into a single compact structure, while enabling dynamic tuning of its operating band. By incorporating varactors or switching elements, the filtenna can adapt its passband in real time. As a result, it provides enhanced spectrum agility and interference suppression for cognitive radio systems. In the following subsections, the design of the reconfigurable filter is first presented and analyzed. Subsequently, this filter is integrated with the antenna to form the complete frequency-reconfigurable filtenna structure.

### Frequency reconfigurable C-shaped filter

**Fig. 14:**
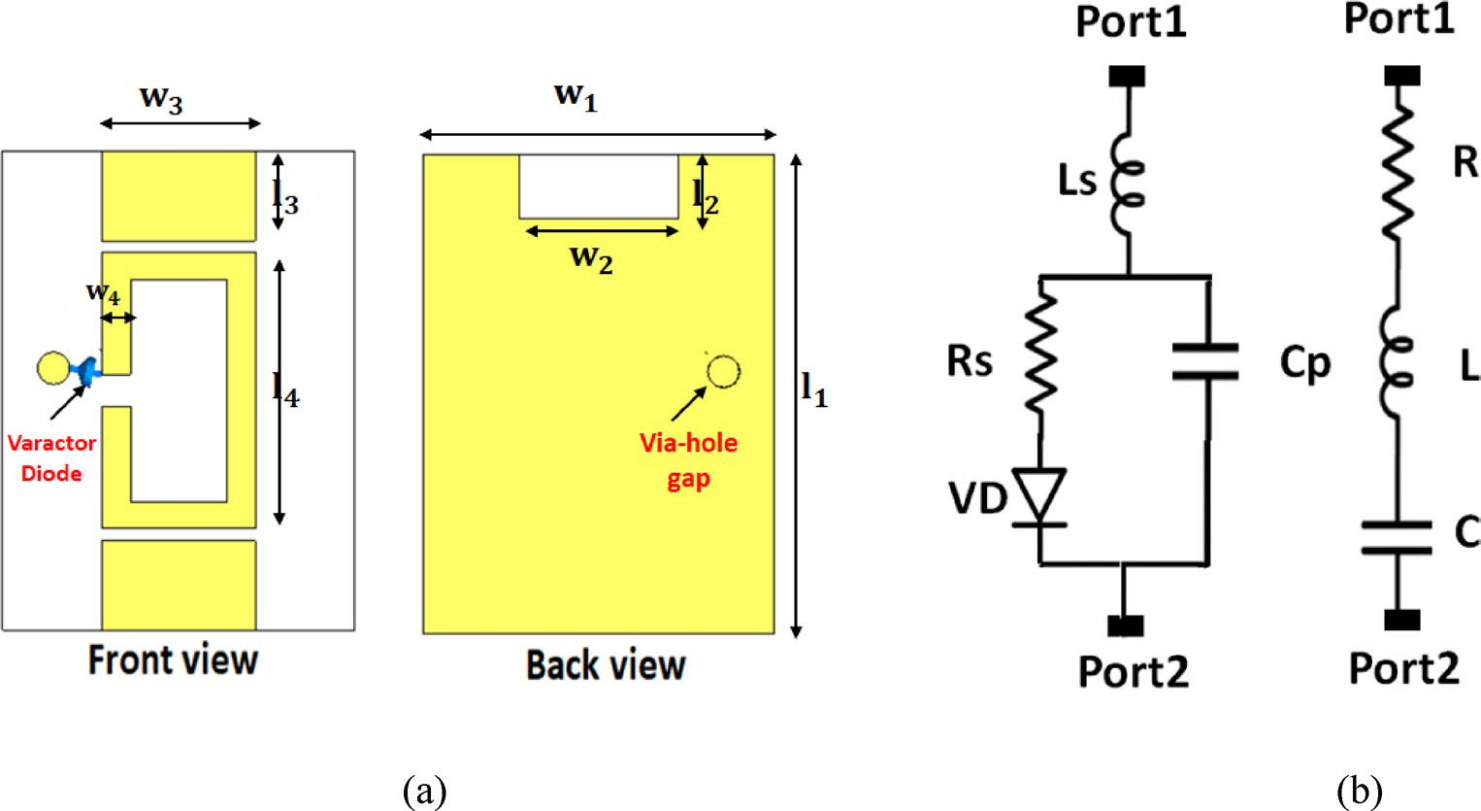
Proposed frequency-reconfigurable filter. (**a**) Front and back views of the filter and (**b**) equivalent electrical circuit model of the varactor-loaded reconfigurable element.

The detailed geometry of the proposed tunable filter is illustrated, as shown in Fig. 14a. A single varactor diode is strategically integrated across hole-gap to provide continuous frequency reconfigurability with minimal biasing complexity and reduced insertion loss. The dimensions of the resonator are optimized for compactness and listed in Table 3. To accurately model the tuning behavior of the varactor, its equivalent circuit is extracted from the manufacturer’s datasheet. In parallel, an ADS-based equivalent model is constructed using parameter-extraction and tuning tools, as shown in Fig. 14b. This ensures that the extracted RLC values match the measured S-parameters of the actual device. The extracted RLC parameters of the employed varactor diode under different reverse-bias voltages ${V}_{R}$, as summarized in Table 4. By adjusting the varactor capacitance from $9.01\:{\mathrm{pF}}$ down to $0.55\:{\mathrm{pF}}$ enables a clear and well-controlled shift in the resonance frequency. This demonstrates a wide continuous tuning range extending approximately from $2.4\:{\mathrm{GHz}}$ up to $3.5\:{\mathrm{GHz}}$, depending on the biasing condition. The filter maintains good matching across all tuning states, with ${S}_{11}\:<\:-10\:{\mathrm{dB}}$ for every resonant point. Consequently, the structure provides a stable tunable bandwidth and a smooth tuning profile. Thereby, it is highly suitable for integration with the following reconfigurable filtenna stage.

**Fig. 15:**
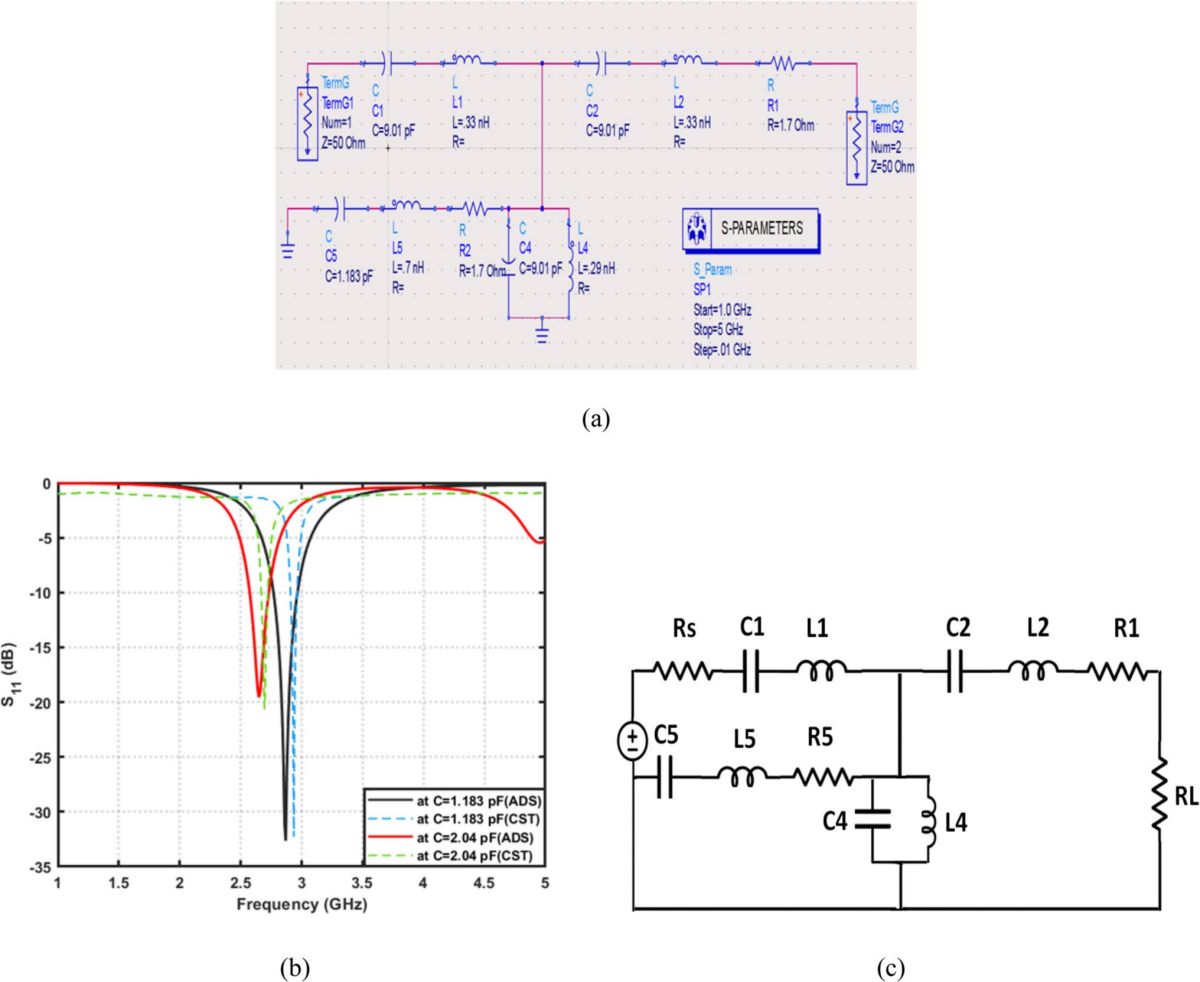
Circuit modeling and frequency response of the proposed tunable filtering structure. (**a**) ADS schematic of the equivalent lumped-element circuit, (**b**) simulated $\left|{S}_{11}\right|$​ response for different capacitance values, and (**c**) Simplified equivalent RLC circuit representation of the proposed tunable filtering network.

Complete ADS schematic model of the proposed reconfigurable filter is presents, as shown in Fig. 15a. The varactor diode is represented using its extracted RLC equivalent parameters at different biasing voltages. The circuit includes series inductors, capacitors, and resistive losses that accurately emulate the distributed behavior of the physical layout. This model enables precise prediction of the filter’s tuning response when the varactor capacitance changes according to Table 4. The schematic clearly shows how the series and shunt resonant branches interact to produce the tunable notch behavior. Figure 15b presents a comparison between the simulated ${S}_{11}$ results from ADS and the full-wave CST simulation for two capacitance states ($C\:=\:1.183\:{\mathrm{pF}}\:\mathrm{a}\mathrm{n}\mathrm{d}\:C\:=\:2.04\:{\mathrm{pF}}$). An excellent agreement is observed between both tools, confirming the accuracy of the parameter extraction and the reliability of the simplified equivalent circuit. The resonant frequency shifts toward higher frequencies as the varactor capacitance decreases, validating the tunable nature of the filter. Moreover, the return-loss levels remain consistently below − 15 dB for all tuning states. Figure 15c shows the final lumped-element equivalent circuit diagram of the reconfigurable filter. All contributing components, series resistance, resonator inductors, capacitors, and the varactor branch, are represented in their optimized topology. This simplified layout highlights the physical interpretation of each resonator path inside the structure. Furthermore, it illustrates how the varactor-loaded branch controls the tuning capability. The model also includes the load impedance RL, ensuring the circuit reproduces practical conditions.

**Table 3:** Optimized geometrical dimensions of the proposed filter.

Parameters	Dimensions (mm)	Parameters	Dimensions (mm)
${\mathrm{w}}_{1}$	11	${\mathrm{w}}_{3}$	5
${\mathrm{l}}_{1}$	15	${\mathrm{l}}_{3}$	3.2
${\mathrm{w}}_{2}$	5	${\mathrm{w}}_{4}$	1
${\mathrm{l}}_{2}$	2	${\mathrm{l}}_{4}$	8.6

**Table 4:** Extracted equivalent circuit parameters of the varactor diode at different reverse bias voltages.

${\mathbf{V}}_{\mathbf{R}\:}$(V)	*R* (Ω)	L (nH)	C (pF)
$0$	1.7	0.7	9.01
1	1.7	0.7	4.09
2	1.7	0.7	2.04
3	1.7	0.7	1.18
4	1.7	0.7	0.81
8	1.7	0.7	0.55

### Integrating of UWB antenna with frequency reconfigurable filter

**Fig. 16:**
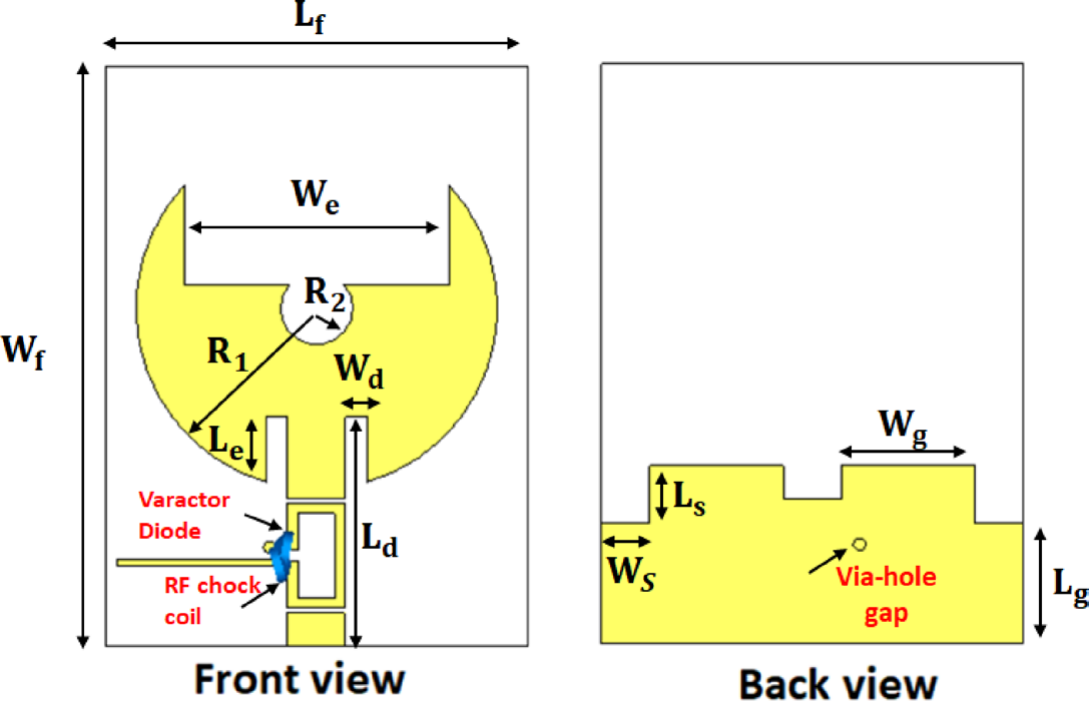
Proposed frequency-reconfigurable filtenna.

**Fig. 17:**
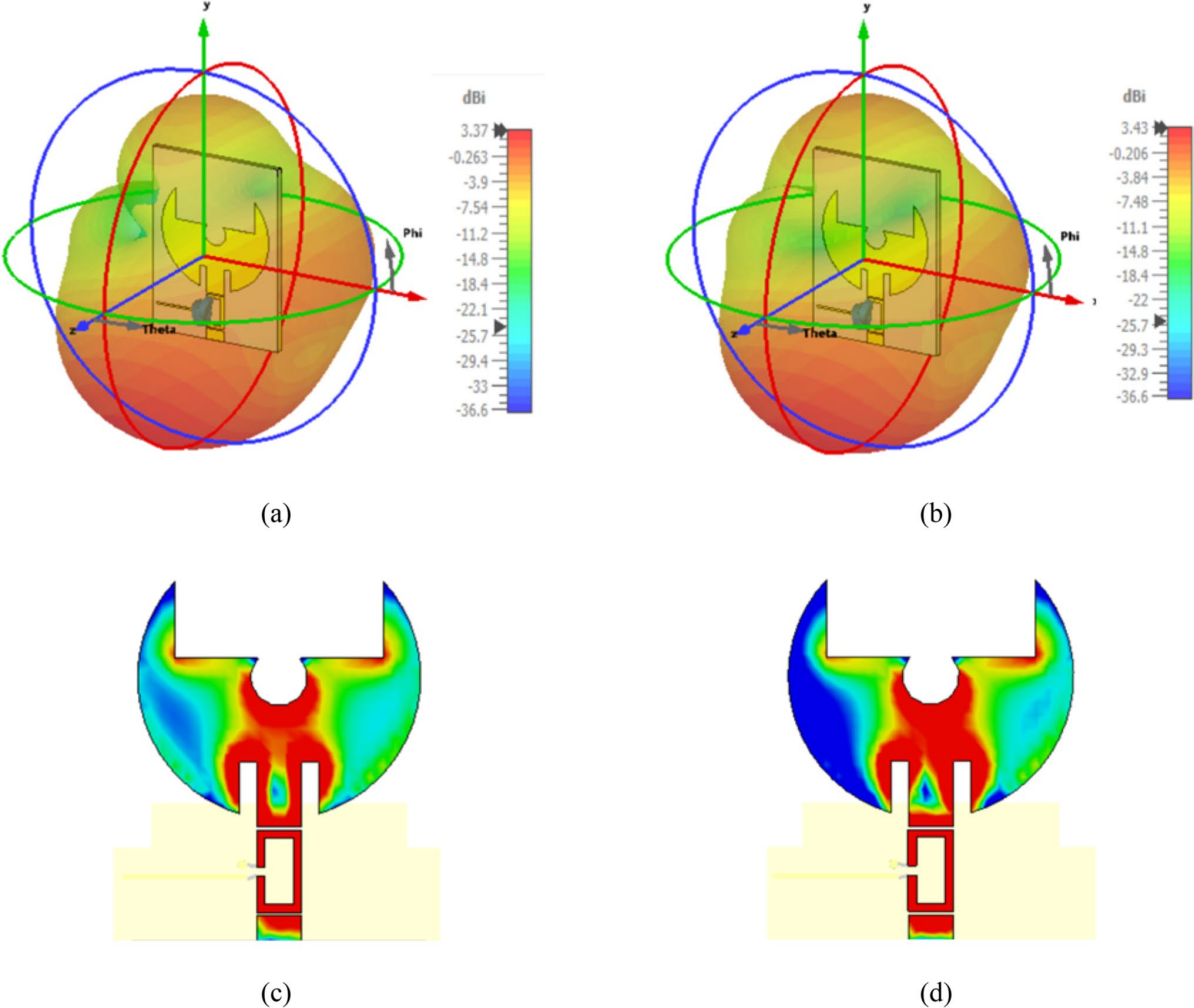
Radiation characteristics and surface current distribution of the proposed frequency-reconfigurable filtenna. (**a**) 3D far-field radiation pattern at $f=3.22\:{\mathrm{GHz}}$, (**b**) 3D far-field radiation pattern at $f=2.95\:{\mathrm{GHz}}$, (**c**) surface current distribution at $f=3.22\:{\mathrm{GHz}}$, and (**d**) surface current distribution at $f=2.95\:\:{\mathrm{GHz}}$.

The proposed reconfigurable filtenna employs a substantially modified radiating patch compared to the initial design. in order to enhance the impedance behavior and accommodate the varactor-based tuning mechanism. As illustrated in Fig. 16, both the geometry of the radiating element and the ground plane have been carefully reshaped to improve impedance matching across the tuning range. Also, the ground structure is reconfigured with stepped slots. Moreover, the feed is implemented using an inset-feed technique to provide finer control of the input impedance and ensure better ${S}_{11}$ performance. To enable frequency reconfigurability, a single varactor diode is integrated across a precisely designed slot. On the other hand, the biasing line is arranged to behave as a high-impedance path at RF frequencies. It prevents unwanted loading of the radiating element. Additionally, an RF choke coil is incorporated in the DC path to block RF currents and isolate the bias network from the RF signal. For clarity and reproducibility, all geometrical dimensions of the proposed filtenna, with modified patch, the feed inset position, and the redesigned ground plane are provided in Table 5. These integrated structural and biasing enhancements collectively contribute to achieve improved ${S}_{11}$ characteristics, higher realized gain, and better radiation efficiency, as listed in Table 6. The 3D radiation characteristics of the proposed frequency-reconfigurable filtenna at two tuning states $3.22\:{\mathrm{GHz}}$ and $2.95\:{\mathrm{GHz}}$ is introduced, as shown in Fig. 17a, b, respectively. In both cases, the antenna exhibits a stable and well-formed radiation envelope. In addition to, Fig. 17c, d illustrate the surface current distribution at the same frequencies. At $C\:=\:1.18\:{\mathrm{pF}}$, the current is densely concentrated around the central slot and the inner edge of the radiating patch. As a result, an extended effective electrical length and therefore a lower resonant frequency are obtained. In contrast, at $C\:=\:0.81\:{\mathrm{pF}}$, the current spreads more uniformly over the patch. This reduces the effective capacitance and shifts the resonant frequency upward. These distinct current paths provide clear physical insight into the tuning mechanism. Also, it validates the role of the varactor diode in controlling the antenna’s resonant behavior.

**Table 5:** Optimized geometrical dimensions of the proposed filtenna.

Parameters	Dimensions (mm)	Parameters	Dimensions (mm)
${\mathrm{w}}_{\mathrm{f}}$	48	${\mathrm{w}}_{\mathrm{g}}$	**11**
${\mathrm{l}}_{\mathrm{f}}$	**35**	${\mathrm{l}}_{\mathrm{g}}$	**10**
${\mathrm{w}}_{\mathrm{e}}$	**22**	${\mathrm{w}}_{\mathrm{s}}$	**4**
${\mathrm{l}}_{\mathrm{e}}$	5.4	${\mathrm{l}}_{\mathrm{s}}$	4.7
${\mathrm{w}}_{\mathrm{d}}$	1.8	${\mathrm{R}}_{1}$	**15**
${\mathrm{l}}_{\mathrm{d}}$	**19**	${\mathrm{R}}_{2}$	3

Significant values are in bold.

**Table 6:** Performance parameters of the proposed frequency-reconfigurable filtenna for different varactor capacitance values.

C (pF)	$\left|{\boldsymbol{S}}_{11}\right|$(dB)	f (GHz)	Efficiency (%)	Gain (dBi)
$9.01$	− 46	2.45	68.5	1.8
4.09	− 38.3	2.58	*70*	2.3
2.04	− 45.5	2.75	*70*	2.84
1.18	− 14	2.95	*73*	3.43
0.81	− 15.2	3.22	*73*	3.37
*0.55*	− 22.4	3.5	68.5	2.3

### Fabrication and measurement setup

**Fig. 18:**
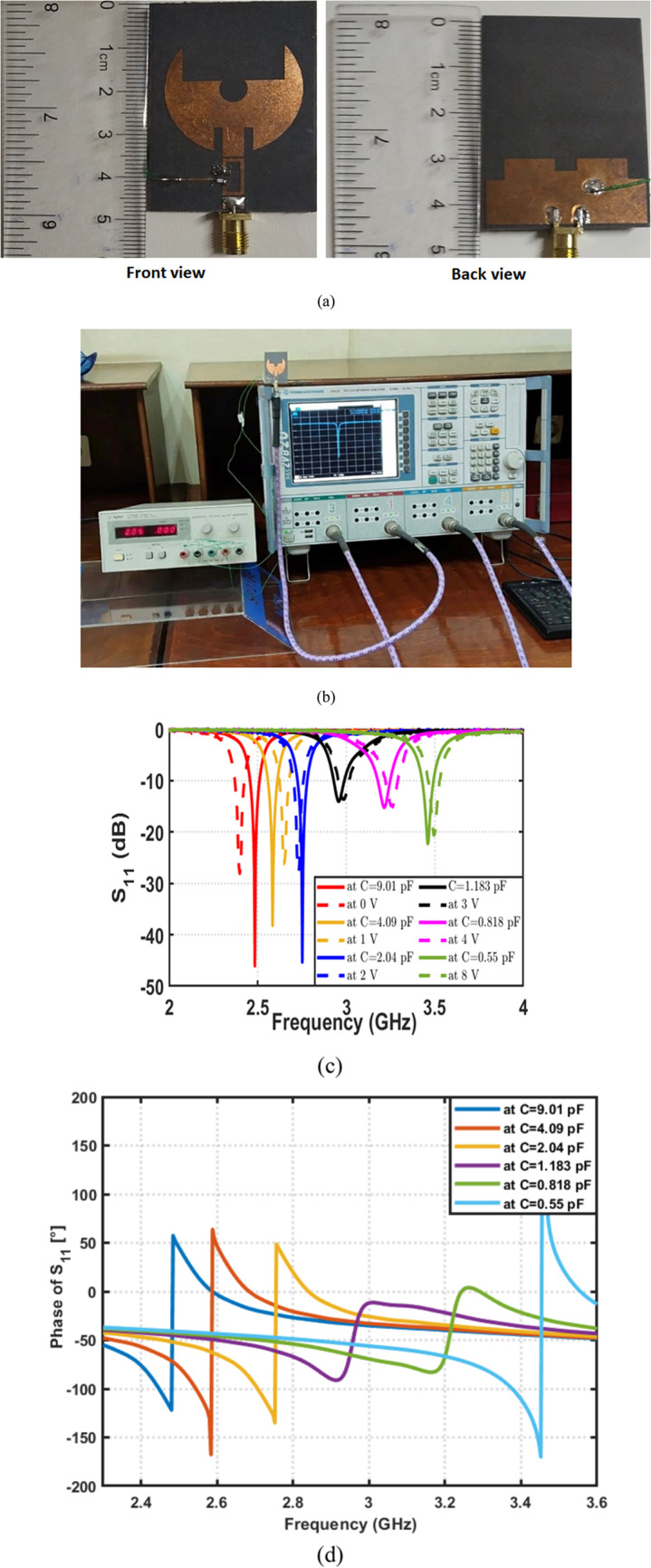
Fabrication, measurement setup, and frequency reconfiguration of the proposed filtenna. (**a**) Photograph of the fabricated prototype, (**b**) Experimental measurement setup, and (**c**) Comparison between measured and simulated reflection coefficients $\left|{S}_{11}\right|$under different bias voltages. (**d**) Phase of simulated reflection coefficient $\angle\:{S}_{11}$ for the proposed filtenna.

**Fig. 19:**
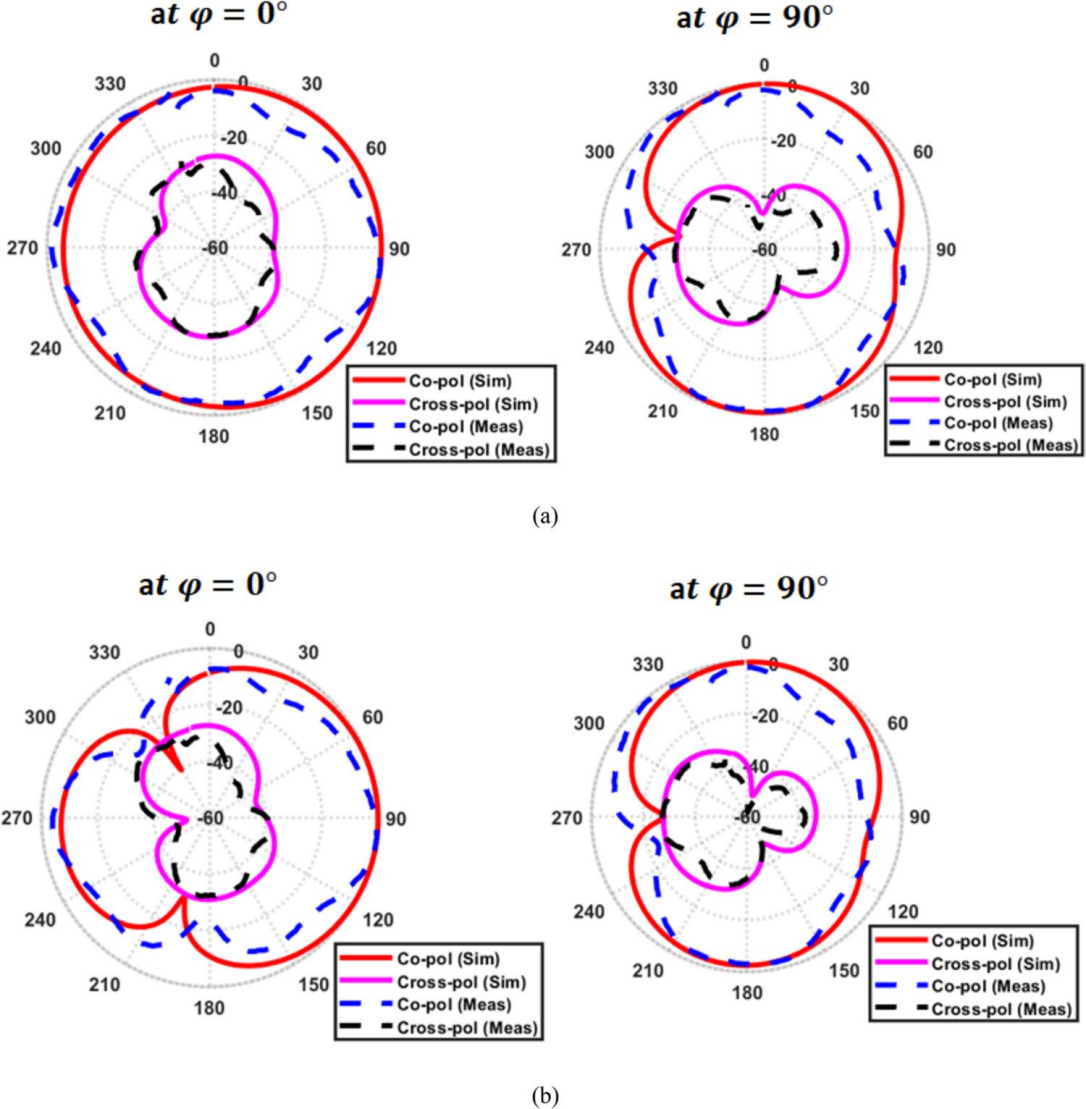
Simulated and measured normalized co- and cross-polarized radiation patterns of the proposed antenna at $\phi\:=0^\circ$ and $\phi\:=90^\circ$ (**a**) at $f=3.22\:{\mathrm{GHz}}$ and (**b**) at $\:f=2.95\:{\mathrm{GHz}}$.

Fabricated prototype of the proposed frequency-reconfigurable filtenna on a Rogers 5880 substrate is presented, as shown in Fig. 18a. Moreover, Fig. 18b presents the complete measurement setup used to evaluate the antenna performance. An Agilent E3620A dual-output DC power supply is used to control the reverse-bias voltage applied to the varactor diode. This enables real-time tuning of the resonance frequency. The filtenna reflection coefficients at different voltage are measured and compared to the simulated ones, as shown in Fig. 18c. It is observed that, as the applied reverse-bias voltage increases, a clear upward shift of the resonance frequency occurs. Also, it is found that the measured results closely follow the simulated trends. Figure 18d presents the reflection phase ($\angle\:{S}_{11}$) of the proposed filtenna for different varactor capacitance values. Unlike the smooth phase variation observed in the standalone UWB antenna (Fig. 4b), the filtenna exhibits a sharp phase transition around the resonance frequency for each tuning state. This abrupt phase change is a clear signature of filtering behavior and confirms the effective integration of the resonant filter within the radiating structure. Moreover, the controlled shift of the phase transition with varying capacitance demonstrates the tunable nature of the filtenna, providing strong evidence that the proposed design operates as a true filtenna rather than a conventional antenna with an attached resonator. Also, the measured polar radiation patterns at the same frequencies for $\phi\:\:=\:0^\circ\:\:\mathrm{a}\mathrm{n}\mathrm{d}\:\phi\:\:=\:90^\circ$ are plotted and compared with the simulated results, as shown in Fig. 19. It can be observed that the measured curves closely follow the simulated ones in both principal planes. A strong dominance of the co-polarized component is observed, with the cross-polarized radiation suppressed by approximately 20–25 dB within the main beam region. Minor deviations between the simulated and measured patterns are observed, which are mainly attributed to fabrication tolerances, soldering effects, and measurement setup imperfections.

## Proposed MIMO frequency reconfigurable antenna

MIMO filtenna has emerged as highly efficient solutions for modern wireless systems, as they integrate both radiation and filtering functionalities into a single compact structure. This integration not only enhances spectrum utilization and reduces front-end losses, but also provides improved isolation, higher selectivity, and better overall system reliability. This makes MIMO filtenna essential for next-generation communication platforms. Therefore, to fully exploit these advantages, $2\times\:2$ MIMO and $4\times\:4$ MIMO filtenna are developed and analyzed, as presented in the following section.

### Proposed $2\times\:2$ MIMO frequency reconfigurable antenna

**Fig. 20:**
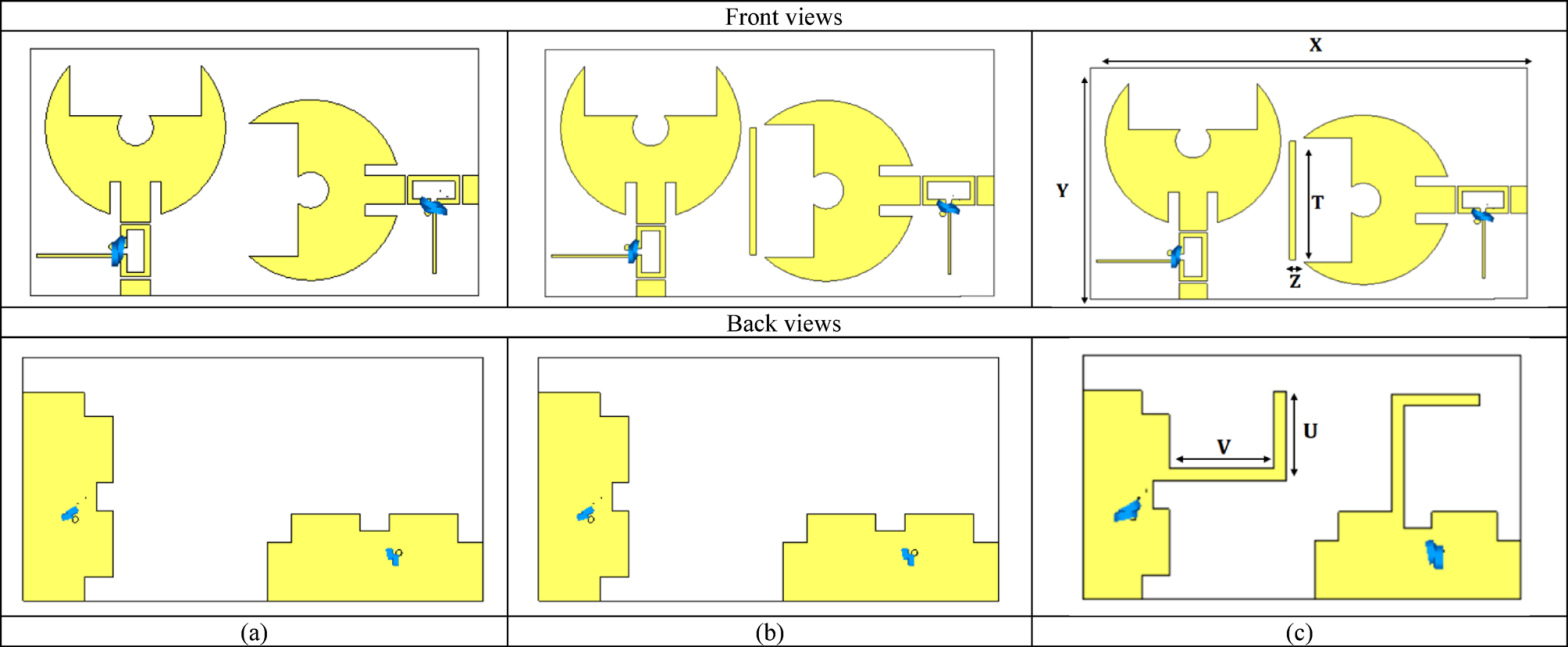
Front and back views of the proposed dual-element filtenna configurations. (**a**) basic structure, (**b**) intermediate decoupling structure, and (**c**) optimized structure with dimensional parameters.

**Fig. 21:**
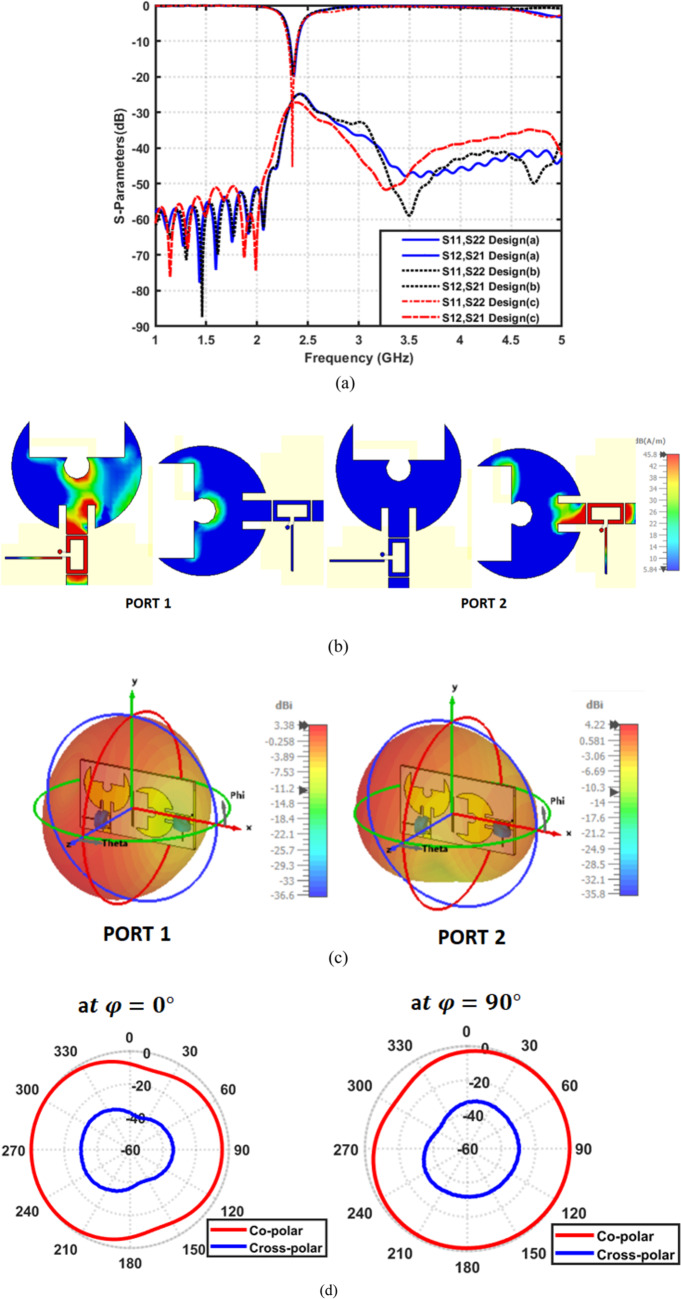
Simulated performance of the proposed 2 × 2 MIMO filtenna at $f=2.45\:{\mathrm{GHz}}$. (**a**) S-parameters ($\left|{S}_{11}\right|$, $\left|{S}_{22}\right|$, and mutual coupling) for different design stages, (**b**) surface current distributions at Port 1 and Port 2, (**c**) 3D radiation patterns for both ports, and (**d**) simulated normalized co- and cross-polarized radiation patterns at ϕ = 0° and ϕ = 90°.

The design procedure of the proposed MIMO filtenna evolved through several refinement stages, as illustrated in Fig. 20. The initial conventional configuration is used as a baseline structure, as shown in Fig. 20a. In the second iteration, an additional inter-element connecting line is introduced to improve mutual coupling suppression and enhance the overall MIMO radiation characteristics, as shown in Fig. 20b. Subsequently, an L-shaped extension is integrated into the ground plane, as shown in Fig. 20(c). It provides an effective current-guiding path that further improves impedance matching, radiation behavior, and key MIMO performance metrics. The final optimized geometry represents the best trade-off between bandwidth reconfigurability and diversity performance, and its corresponding dimensional parameters are summarized in Table 7. Figure 21a compares the simulated reflection coefficients of the initial design stages with the final proposed design at $2.45\:{\mathrm{GHz}}$. The design (a) exhibits a relatively limited tuning behavior with a single dominant resonance and moderate return-loss performance. After introducing the inter-element line modification in design (b), the impedance matching improves and the resonance becomes deeper and more stable. However, noticeable fluctuations in $\left|{\mathrm{S}}_{11}\right|$​ still appear at higher frequencies, indicating that further optimization is required. In contrast, the proposed design shows a significantly enhanced matching response, characterized by a much deeper $\left|{\mathrm{S}}_{11}\right|$​ ​ notch and a wider effective bandwidth. Beside, Fig. 21b presents the simulated surface current distribution of the proposed 2 × 2 reconfigurable MIMO filtenna at 2.45 GHz. As shown, the clear current confinement on each radiating patch with minimal current leakage toward adjacent elements indicates excellent port isolation, consistent with the low measured ECC. Figure 21c illustrates the 3D realized gain radiation pattern of the proposed MIMO filtenna operating at 2.45 GHz. To further illustrate the radiation characteristics of the proposed antenna in the principal planes, normalized 2D co- and cross-polarized polar radiation patterns are presented at $\phi\:\:=\:0^\circ\:\:\mathrm{a}\mathrm{n}\mathrm{d}\:\phi\:\:=\:90^\circ\:,$ as shown in Fig. 21d. The results clearly indicate the dominance of the co-polarized components. Table 8 summarizes the radiation characteristics of the proposed 2 × 2 MIMO filtenna across different reverse-bias voltages applied to the varactor diode. The isolation values remain consistently better than − 20 dB across all tuning states, confirming excellent mutual-coupling suppression between the two MIMO elements. Additionally, the radiation efficiency remains relatively high, varying between 74.5 and 81.6%, which verifies that integrating the varactor and its biasing network does not significantly degrade the antenna’s radiation characteristics. The realized gain shows stable performance across the tuning range, increasing from 3.32 dBi at 2.75 GHz to 4.57 dBi at 3.48 GHz. This gradual gain enhancement with increasing frequency aligns with the natural behavior of electrically small antennas operating at higher frequencies.

**Fig. 22:**
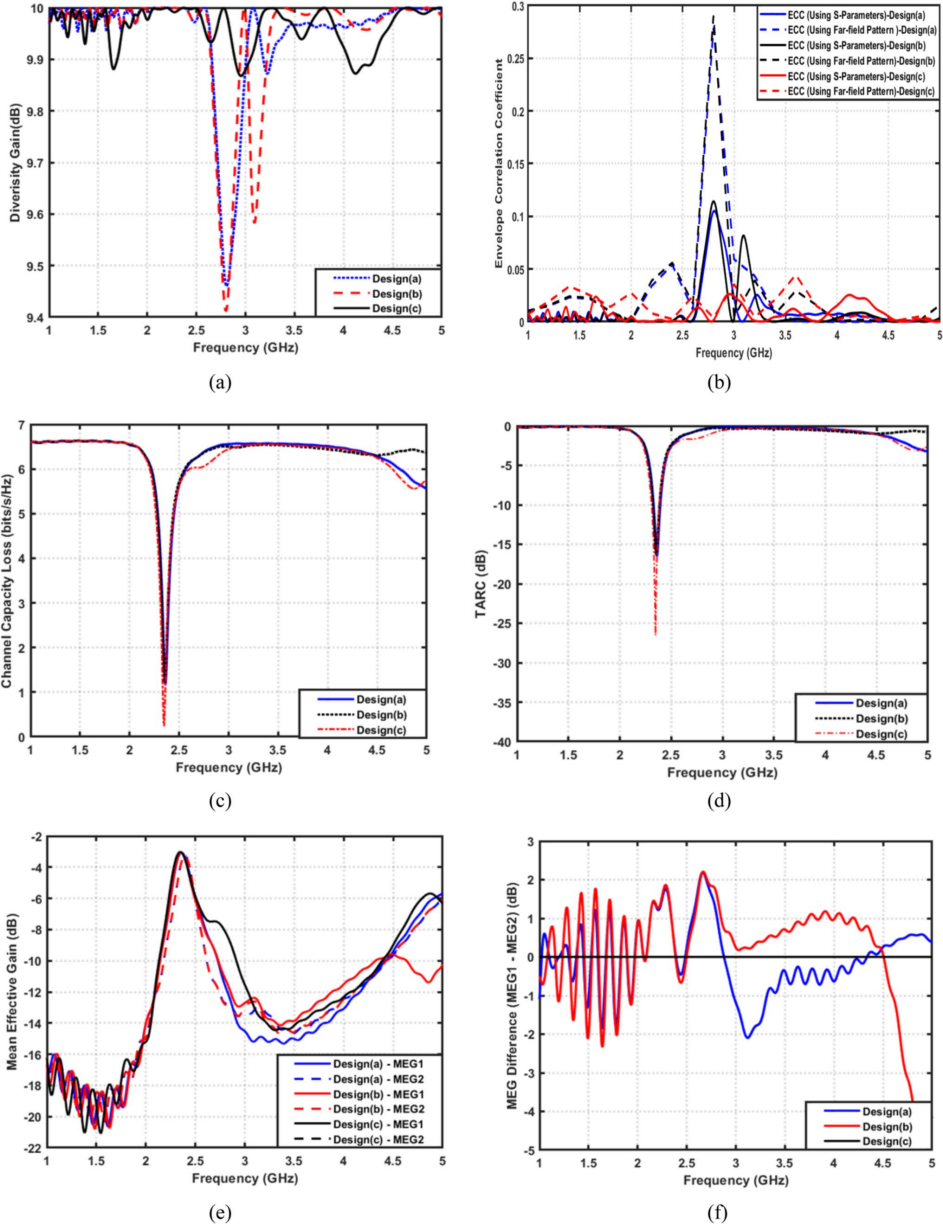
2 × 2 MIMO performance evaluation of the proposed filtenna configurations. (**a**) DG, (**b**) ECC, (**c**) CCL, (**d**) TARC, (**e**) MEG for both filtenna elements, and (**f**) MEG difference versus frequency.

Figure 22a presents the diversity gain for the proposed MIMO configurations at $2.45\:{\mathrm{GHz}}$. Design (a) exhibits a pronounced DG degradation due to strong inter-element coupling. Design (b) partially alleviates this issue. However, the deep DG dip remains visible. In contrast, the final proposed design sustains a DG value very close to the ideal $10\:{\mathrm{dB}}$ across the entire operational band, with substantially reduced fluctuations. This confirms that the modified radiating structure and engineered ground plane significantly enhance diversity performance and minimize coupling-induced losses. Also, Fig. 22(b) illustrates the envelope correlation coefficient evaluated using both S-parameter-based and radiation-pattern-based approaches for three different design stages. It is observed that Design (a) exhibits relatively high ECC values, particularly around the resonant frequency, indicating strong mutual coupling and highly correlated radiation behavior. After introducing partial structural modifications, Design (b) shows an improvement in ECC; however, noticeable correlation peaks still persist, especially when ECC is extracted from the far-field radiation patterns, revealing residual radiation coupling between antenna elements. In contrast, the proposed Design (c) demonstrates the lowest ECC values across the investigated frequency range using both evaluation methods. More importantly, the radiation-based ECC remains consistently below 0.05, confirming true radiation diversity and weak correlation between the antenna elements. The close agreement between the S-parameter-based and far-field-based ECC curves in Design (c) further validates the robustness of the proposed decoupling strategy. This behavior confirms that the implemented structural and ground-plane modifications effectively suppress both surface-wave and radiation coupling, leading to highly independent radiation characteristics. On the other hand, Fig. 22c illustrates the channel capacity loss. Both design (a) and design (b) show noticeable performance reduction around the resonant frequency. The proposed design achieves the lowest CCL, maintaining values tightly clustered near the theoretical limit and consistently below the acceptable threshold. This confirms the ability of the proposed antenna to preserve high MIMO channel capacity despite variation in frequency and coupling effects. Additionally, Fig. 22d reports the total active reflection coefficient (TARC) for the considered designs. Design (a) and Design (b) exhibit relatively deeper mismatch around 2.4 GHz, with TARC minima reaching approximately − 15 dB and − 18 dB, respectively, indicating higher sensitivity to multi-port excitation conditions. In contrast, the proposed design (Design (c)) achieves the lowest TARC value, reaching approximately $-25\:{\mathrm{dB}}$ at resonance. This behavior indicates a more favorable impedance environment, improved excitation balance among the MIMO elements, and enhanced robustness against simultaneous multiport excitations with varying signal phases and amplitudes. Figure 22e shows the mean effective gain (MEG) for each antenna element. Designs (a) and (b) exhibit degraded MEG, reflecting less stable performance in real multipath environments. The proposed design demonstrates smoother MEG behavior and higher values. Furthermore, Fig. 22f presents the MEG difference between the two antenna elements. Design (a) exhibits large oscillations and significant discrepancies across the band. Design (b) reduces the oscillatory behavior, but still displays considerable imbalance. The proposed design closely follows the ideal zero-difference line, indicating nearly identical power reception characteristics for both MIMO elements.

**Table 7:** Optimized geometrical dimensions of the proposed 2 × 2 MIMO filtenna structure.

Parameters	Dimensions (mm)	Parameters	Dimensions (mm)
$\mathrm{X}$	75	$\mathrm{T}$	21
$\mathrm{Y}$	40	$\mathrm{U}$	15
$\mathrm{Z}$	1	$\mathrm{V}$	18

**Table 8:** Performance of the proposed 2 × 2 MIMO filtenna under different varactor capacitance values.

C (pF)	Isolation (dB)	f (GHz)	Efficiency (%)	Gain (dBi)
9.01	− 27.5	2.45	81.6	4.22
4.09	− 24.5	2.58	81	3.46
2.04	− 22	2.75	80	3.32
1.18	− 21	2.95	*78*	3.51
0.81	− 20.5	3.2	75	4.29
0.55	− 23	3.48	74.5	4.57

### Proposed × MIMO frequency reconfigurable filtenna

**Fig. 23:**
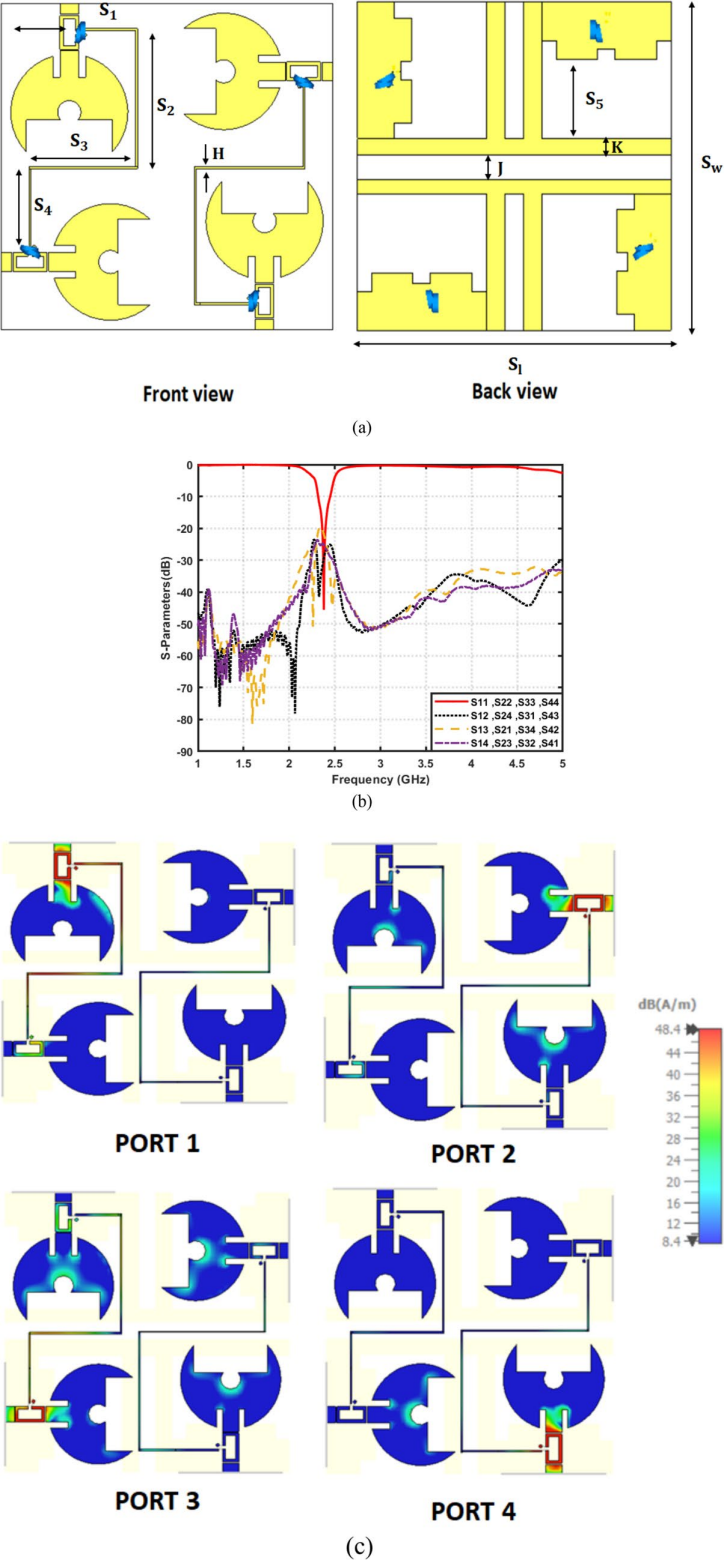

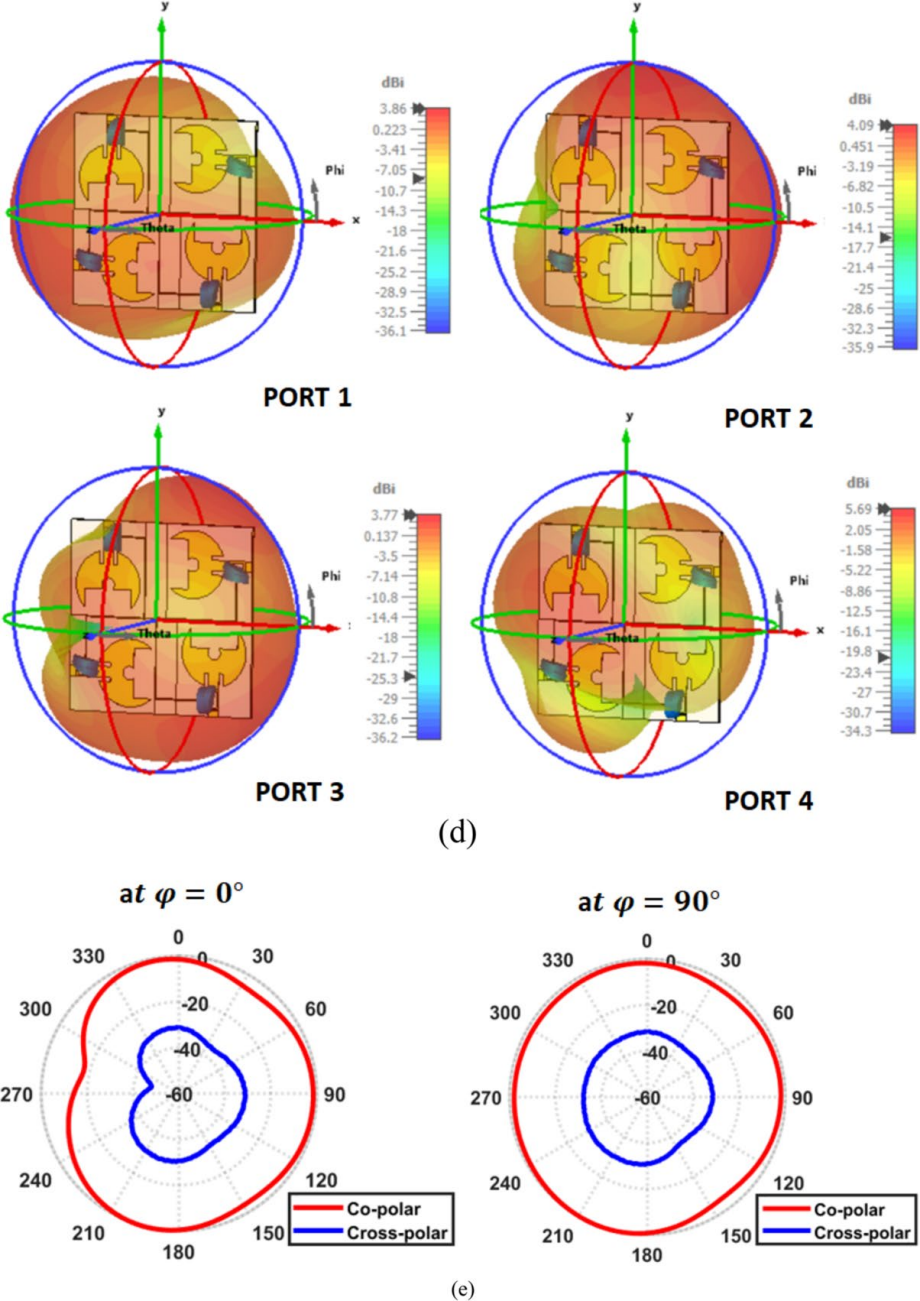
Proposed quad-element filtenna. (**a**) Basic structure, (**b**) S-parameters ($\left|{S}_{11}\right|$, $\left|{S}_{22}\right|$, and mutual coupling), (**c**) surface current distributions at all ports, (**d**) 3D radiation patterns for all ports, and (**e**) simulated normalized co- and cross-polarized radiation patterns at ϕ = 0° and ϕ = 90°.

To further validate the scalability and robustness of the proposed architecture, a 4 × 4 MIMO frequency-reconfigurable filtenna array is also developed, as shown in Fig. 23a. The values of ${\mathrm{S}}_{\mathrm{l}\:}=85{\:{\mathrm{mm}},\:\mathrm{S}}_{\mathrm{w}}=84\:\mathrm{m}\mathrm{m}\:,\:{\mathrm{S}}_{1}=13\:\mathrm{m}\mathrm{m}\:,\:{\mathrm{S}}_{2}=35\:\mathrm{m}\mathrm{m}\:,\:{\mathrm{S}}_{3}=27.5\:\mathrm{m}\mathrm{m}\:,\:{\mathrm{S}}_{4}=21\:\mathrm{m}\mathrm{m}\:,{\mathrm{S}}_{5}=20\:{\mathrm{mm}},\mathrm{H}\:=0.5\:\mathrm{m}\mathrm{m},\:\:\mathrm{J}\:=6.4\:\mathrm{m}\mathrm{m},$and $\mathrm{K}\:=4\:\mathrm{m}\mathrm{m}$ are annotated on the illustrated layout for clarity. In the proposed design, a pairwise DC-biasing strategy is implemented, where each two adjacent antenna elements share a common biasing line. This configuration significantly simplifies the biasing layout while preserving high RF integrity. The shared biasing line feeding each antenna pair is designed with a high-impedance path. This guarantees that the DC control voltage reaches both varactor diodes without allowing RF energy to leak into the bias network. This pairwise arrangement also reduces routing congestion, minimizes parasitic loading, and avoids the strong inter-element coupling. Additionally, the ground plane is modified to incorporate a $\pi$-shaped interconnecting ground section positioned between each pair of antenna grounds. This localized ground provides a controlled return path that reduces mutual coupling. Figure 23b presents the simulated S-parameters of the proposed 4 × 4 frequency-reconfigurable MIMO filtenna at $2.45\:{\mathrm{GHz}}.$ The reflection coefficients exhibit deep resonances, confirming excellent impedance matching across all ports. Meanwhile, the mutual-coupling remain well below $-20\:{\mathrm{dB}}$. It demonstrates the effectiveness of the adopted isolation enhancing techniques. Moreover, Fig. 23c illustrates the surface-current distribution for each excited port at 2.45 GHz. When one port is fed, the current remains highly concentrated around the corresponding radiating patch, varactor region, and its associated feed structure. While, only minimal induced currents appear on the remaining elements. This confirms the low inter-element coupling observed in the S-parameter results. In addition, Fig. 23d displays the 3D pattern of the full 4 × 4 MIMO filtenna at 2.4 GHz. Corresponding normalized 2D polar cuts in the principal planes are also shown in Fig. 23e. The results reveal that the radiated field is primarily governed by the co-polarized component, whereas the cross-polarized contribution is markedly reduced. This behavior reflects well-controlled polarization characteristics and indicates reliable radiation performance. Table 9 summarizes the key performance indicators of the proposed 4 × 4 frequency-reconfigurable MIMO filtenna under different varactor capacitance values. As the capacitance decreases from $9.01\:\mathrm{t}\mathrm{o}\:0.55\:\mathrm{p}\mathrm{F}$, a clear upward tuning in the resonant frequency is observed, shifting from$\:2.45\:\mathrm{G}\mathrm{H}\mathrm{z}\:\mathrm{u}\mathrm{p}\:\mathrm{t}\mathrm{o}\:3.48\:\mathrm{G}\mathrm{H}\mathrm{z}$. Across all tuning states, the antenna maintains excellent inter-element isolation, ranging from − 16 to − 33 dB, which verifies the robustness of the implemented ground modification and decoupling techniques in the 4-port configuration. Moreover, the radiation efficiency remains high, between 71.8 and 74%, demonstrating that the integration of the varactor diode and biasing network does not compromise antenna performance. The realized gain varies between $3.8\:\mathrm{d}\mathrm{B}\mathrm{i}\:\mathrm{a}\mathrm{n}\mathrm{d}\:4.95\:\mathrm{d}\mathrm{B}\mathrm{i}$, showing stable radiation performance across the tuning range, with peak gain occurring at the mid-capacitance state ($1.183\:\mathrm{p}\mathrm{F}$).

**Fig. 24:**
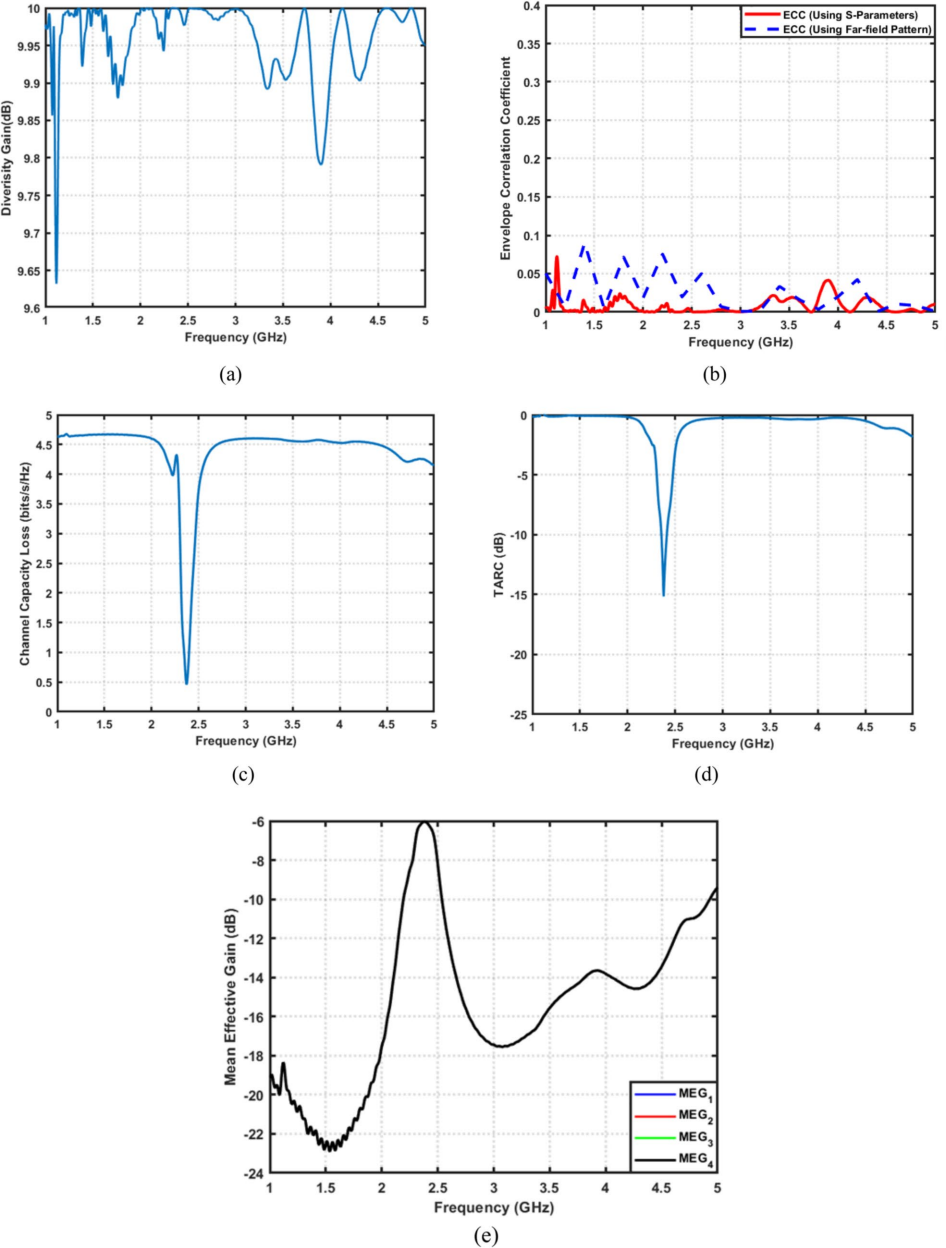
4 × 4 MIMO performance evaluation of the proposed filtenna configuration. (**a**) DG, (**b**) ECC, (**c**) CCL, (**d**) TARC, and (**e**) MEG for all filtenna elements.

The proposed MIMO design is thoroughly evaluated at $2.45\:\mathrm{G}\mathrm{H}\mathrm{z}$ in terms of its key MIMO performance parameters, as shown in Fig. 24. The ECC performance of the proposed 4 × 4 MIMO filtenna using both extraction techniques is presented. Despite the increased number of antenna elements and the compact integration of the filtering and tuning circuitry, the radiation-based ECC remains consistently low within the operational band around 2.45 GHz. Compared to the S-parameter-based results, the radiation-based ECC reveals slightly higher peaks, reflecting a more realistic assessment of pattern correlation under multiport excitation. Nevertheless, the ECC values remain significantly below the threshold required for efficient MIMO operation. This validates the effectiveness of the proposed isolation structures and confirms excellent diversity performance even in the higher-order MIMO configuration. The DG approaches the ideal value of $\sim10\:\mathrm{d}\mathrm{B}$. The channel capacity loss is significantly low, staying far below the $0.5\:\mathrm{b}\mathrm{i}\mathrm{t}\mathrm{s}/\mathrm{s}/\mathrm{H}\mathrm{z}$ threshold. The TARC profile reveals a significant reduction around 2.45 GHz, reaching approximately $-15\:\mathrm{d}\mathrm{B}$, which confirms favorable active impedance conditions during multiport excitation. In addition, the consistently low TARC levels $(\le\:\:-10\:\mathrm{d}\mathrm{B})$ across the band highlight the robustness of the proposed MIMO configuration against excitation imbalance. Meanwhile, The MEG values for the four ports remain well-balanced, ensuring uniform antenna behavior, which verifies high MIMO efficiency. In conclusion, this extended configuration demonstrates that the proposed filtenna concept can be effectively expanded to higher-order MIMO systems while preserving stable impedance matching, high isolation, and consistent reconfigurable performance across all ports.

To further justify the scalability of the proposed architecture from a single UWB antenna to reconfigurable filtenna and multiport MIMO filtenna configurations, the quality factor $Q=\frac{{f}_{0}}{BW}$ ​​ is evaluated and summarized in Table 10. As expected, the single UWB antenna exhibits a very low $Q$ value$\:\left(\approx\:\:0.93\right)$, which is characteristic of ultra-wideband radiators with inherently large fractional bandwidths. When the antenna is transformed into a frequency-reconfigurable filtenna, the bandwidth becomes intentionally constrained to achieve filtering and spectral selectivity. This leads to a substantial increase in the quality factor ($Q\approx\:42.9$). This behavior confirms the classical bandwidth–$Q$ trade-off, where enhanced selectivity and filtering functionality are achieved at the expense of bandwidth. Extending the filtenna to 2 × 2 and 4 × 4 MIMO configurations introduces additional electromagnetic coupling, shared ground structures, and biasing networks. As a consequence, the effective bandwidth is reduced, leading to increased quality factors of approximately 38.3 and 45.4 for the 2 × 2 and 4 × 4 MIMO filtenna configurations, respectively. Despite this increase in Q, the proposed MIMO filtenna architectures maintain stable impedance matching, high radiation efficiency, and excellent diversity performance. These results demonstrate that the proposed design framework follows a physically consistent and theoretically justified evolution.

**Table 9:** Performance of the proposed 4 × 4 MIMO Filtenna under different varactor capacitance values.

C (pF)	Isolation (dB)	f (GHz)	Efficiency (%)	Gain (dBi)
9.01	− 23	2.45	71.8	3.8
4.09	16	2.58	72.2	4.3
2.04	− 33	2.75	71.8	4.4
1.18	− 30	2.95	72	4.95
0.81	− 26.5	3.2	73	4.75
0.55	− 23	3.48	74	4.24

**Table 10:** Resonant frequency, bandwidth, and quality factor of the proposed antenna configurations.

Configuration	${\boldsymbol{f}}_{0}$ (GHz)	$\boldsymbol{B}\boldsymbol{W}$ (GHz)	$\boldsymbol{Q}$
Single UWB antenna	5.2	5.6	0.93
Single filtenna	3	0.07	42.86
$2\times\:2$ MIMO filtenna	2.45	0.064	38.28
$4\times\:4$ MIMO filtenna	2.45	0 0.054	45.37

### Comparison with related work

A detailed comparison has been conducted, to benchmark the proposed 2 × 2 and 4 × 4 MIMO filtenna architectures against recently published designs, as summarized in Table 11. The results clearly show that the proposed structures outperform prior works across key performance metrics. While several existing antennas exhibit limited tuning bandwidth, moderate isolation, or relatively high ECC values, the proposed designs achieve a broader reconfigurable range with stable gain and high radiation efficiency throughout all tuning states. In comparison, many referenced designs show higher ECC, lower gain, and reduced efficiency, particularly when aiming for compact geometries. The proposed 4 × 4 filtenna achieves a peak gain of 4.95 dBi and efficiency around 74%, which are highly competitive given the compact footprint and the inclusion of varactor-based frequency tuning. On the whole, the comparative analysis highlights that the proposed work achieves a superior balance of compact size, wide tunability, high isolation, and strong MIMO performance.

**Table 11:** Comparative performance analysis of recent MIMO Filtenna designs.

References	Antenna size (mm)	Number of ports	Operating frequency (GHz)	Peak efficiency (%)	Peak gain (dBi)	Isolation (dB)	ECC	Reconfiguration technique/single element
^35^	70 × 70	4	2.5, 3.3, 3.5, 4.7, 5.2	90	1.74	<− 28	< 0.16	Single PIN diode
^36^	20 × 35	2	2.95–3.28, 3.35–3.94, 3.8–4.6, 4.03–5.19	62% at 5 GHz, 75% at 3.5 GHz	2.47	<− 10.2	< 0.09	Single PIN diode
^37^	120 × 60	2	0.8–6	74.5	2.97	<− 10	< 0.26	Two PIN diodes
^38^	48 × 24	2	1.77, 4.75	89.64	4.41	<− 26.52	< 0.253	Single PIN diode
^39^	76 × 44	8	1.29–2.51	85	4.75	<− 28	0.0013	Liquid-dielectric
^40^	120 × 100	4	2.8–3.65	> 70	3	NA	< 0.01	Single varactor
Proposed Work 2 × 2 MIMO	75 × 40	2	2.45, 2.58, 2.75, 2.95, 3.2, 3.48	81.6	4.57	<-27.5	< 0.025	Single varactor
Proposed Work 4 × 4 MIMO	85 × 84	4	2.45, 2.58, 2.75, 2.95, 3.2, 3.48	74	4.95	<-33	< 0.02	Single varactor

## Conclusion

This work presented a comprehensive development of compact wideband antennas, reconfigurable filtenna structures, and advanced MIMO configurations tailored for 5G and cognitive radio systems. A fork-shaped UWB antenna was first designed, optimized, fabricated, and experimentally validated, achieving a wide impedance bandwidth from $2.4\:to\:8\:{\mathrm{GHz}}$ with stable gain and high radiation efficiency. Building on this geometry, 4 × 4 UWB MIMO arrays were developed, demonstrating excellent isolation, very low correlation, and strong diversity performance. A frequency-reconfigurable filtenna was then introduced by integrating a varactor-tuned filter into a modified radiating patch. The fabricated prototype achieved continuous tuning from $2.45\:to\:3.48\:{\mathrm{GHz}}$, with measured results closely matching simulations. To extend system capability, 2 × 2 and 4 × 4 MIMO filtenna architectures were proposed, employing engineered ground structures, decoupling lines, and high-impedance biasing networks to improve isolation and suppress surface-wave coupling. Across all configurations, the antennas exhibited highly stable gain, efficient radiation, and robust tuning characteristics. The MIMO evaluation metrics confirmed excellent diversity performance, strong channel capacity, and minimal correlation. These results clearly demonstrate that the proposed designs overcome key limitations of existing antennas by providing a superior combination of compact integration, wide tunability, high isolation, and strong spectral agility. Consequently, the proposed UWB antenna, reconfigurable filtenna, and their MIMO extensions offer a robust and highly efficient front-end solution for next-generation wireless platforms, including sub-6 GHz 5G networks, dynamic spectrum access, and cognitive radio systems. Future research directions will focus on further optimizing and extending the proposed antenna design. One important direction is the experimental fabrication and measurement of the complete multiport MIMO configuration. Another promising extension involves system-level evaluation, such as channel capacity analysis under dynamic spectrum access scenarios and integration with real-time spectrum sensing algorithms.

## Data Availability

The datasets generated and/or analyzed during the current study are available from the corresponding author **Hager S. Fouda** on reasonable request.
