# Correction: A scalable UWB-to-reconfigurable MIMO filtenna with single-varactor tuning and enhanced isolation for adaptive 5G and cognitive radio systems

**DOI:** 10.1038/s41598-026-47801-2

**Published:** 2026-04-09

**Authors:** Hager S. Fouda, Amal S. Hamoud, Mahmoud A. Attia

**Affiliations:** 1 Electronics and Electrical Communications Engineering Department, Tanta University, Tanta, Egypt; 2 Electronics and Electrical Communications Engineering Department, Faculty of Engineering, Horus University-Egypt, New Damietta, Egypt

Correction to: *Scientific Reports* 10.1038/s41598-026-36882-8, published online 13 February 2026

In the original version of this Article, Amal S. Hamoud was omitted as a corresponding author. Correspondence and requests for materials should also be addressed to: PG_189490@f-eng.tanta.edu.eg

The original Article has been corrected.

